# Patch seriation to visualize data and model parameters

**DOI:** 10.1186/s13321-023-00757-1

**Published:** 2023-09-09

**Authors:** Rita Lasfar, Gergely Tóth

**Affiliations:** Institute of Chemistry, Eötvös Loránd University, Pázmány sétány 1/a, Budapest, 1117 Hungary

**Keywords:** Seriation, Data visualization, Model interpretation, Clustering, Neural network model

## Abstract

We developed a new seriation merit function for enhancing the visual information of data matrices. A local similarity matrix is calculated, where the average similarity of neighbouring objects is calculated in a limited variable space and a global function is constructed to maximize the local similarities and cluster them into patches by simple row and column ordering. The method identifies data clusters in a powerful way, if the similarity of objects is caused by some variables and these variables differ for the distinct clusters. The method can be used in the presence of missing data and also on more than two-dimensional data arrays. We show the feasibility of the method on different data sets: on QSAR, chemical, material science, food science, cheminformatics and environmental data in two- and three-dimensional cases. The method can be used during the development and the interpretation of artificial neural network models by seriating different features of the models. It helps to identify interpretable models by elucidating clusters of objects, variables and hidden layer neurons.

**Graphical Abstract:**

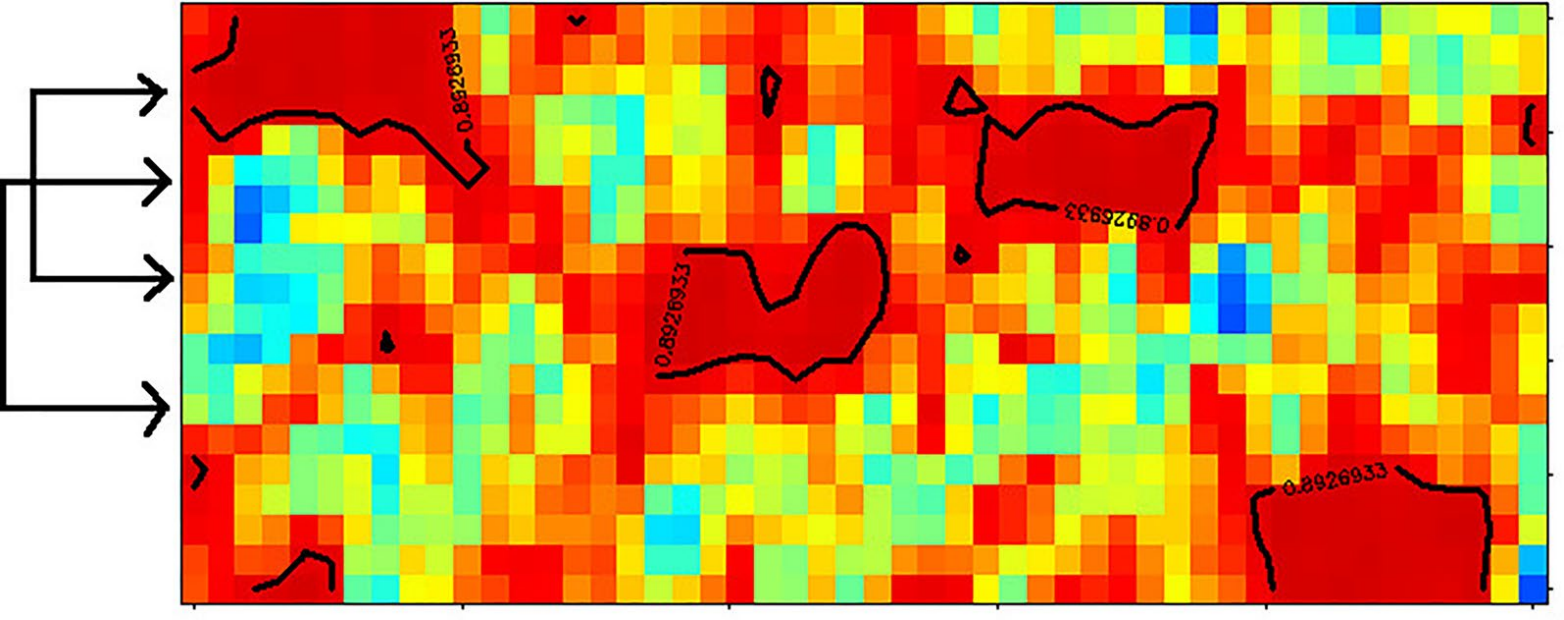

**Supplementary Information:**

The online version contains supplementary material available at 10.1186/s13321-023-00757-1.

## Introduction

Seriation? Most scientists are involved in it without knowing the term. If one knows its practical definition, namely, how to do row and/or column permutations to enhance visual perception of a table or heatmap, it is clear for scientists that they faced the problem. Its first application goes back to the nineteenth century [1], when it was an explanatory technique to order objects in a way to reveal patterns and regular features easily. Later it spread to all fields of science and the ordering often concern two sequences to be reordered [2–5]. There are, e.g., possibilities to order objects along two axes or one object and one variable sequences in a table. The first applications were connected to fields, where visualization or chronological sequence were natural (archaeology, cartography, history, operation research, sociology). Later, especially when information technology was present, different methods and applications appeared in many other fields (anthropology, graphics, information visualization, sociometry, psychology, psychometry, ecology, biology, bioinformatics, etc…). The common feature in the methods is that they are not common for all fields of science. There is a rather small communication among the fields. The most general review was written by Liiv [5], where a historical overview of seriation is detailed including the milestones at several application fields. Instead of enumerating here the methods and provide a deficient and scanty list of applications, we forward the reader to the review of Liiv [5].

Seriation is applied in a latent way in chemistry [6–9], and it is seldom termed. It is often used in many scientific software as a default setting, that, e.g., hierarchical clustering is applied on objects and a visually acceptable sequence is generated using a seriated dendogram [10–13]. The importance of seriation related methods in bioinformatics has led to an increase in the number of special methods and their applications in other fields, as in cheminformatics. From these, we mention only the bi- or co-clustering [14, 15]. From the special applications in bioinformatics, we may refer to similarity search and alignment methods, where our references are some recent reviews. Similarity searching is widely applied and encompasses many techniques, its principle is based on detecting molecules that have the same biological activity [16]. By the same logic, alignment is based on hypothesis of homology [17] (sequential similarity). The real number of alignment algorithms is in hundreds and continue to increase.

Going back to chemistry, we found up to now only a few articles, where the word seriation is used in the title, abstract or in the keywords [18–21]. One interesting example for seriation in chemistry, while not the main subject of the article, showed its importance in summarising the resulting relationships between production groups and chemical clusters, and has enabled external information to be compared with the cluster results [18].

The main aim of seriation is to get better visualization by introducing some order by appropriate permutation of the rows and/or columns. In the literature of seriation, it is often called sequencing. The final sequence may help to find similar objects or variables close to each other in vectors, tables or in their graphics (e.g., in heatmaps). In some cases, it can be the first visual check of data and it serves as a good starting point to estimate which enhanced data analysis method might be tried. Seriation offers the advantage to be a method where all information is kept in the seriated data, only the usually ad hoc original order of rows and columns is changed. The specialized methods may outperform seriation, e.g., clustering is usually more efficient to identify similar objects than seriation. There are several graphical representation ways and performance measures to help the interpretation of clustering results, while the only accessible information of traditional seriation is a visual check of a reordered heatmap. Furthermore, clustering methods might provide object or variable arrangements that cannot be shown by a one-dimensional sequence of objects or variables. The simple hierarchical clustering of objects/variables, bi- and co-clustering methods might be interpreted as one- or two-dimensional seriations, because their results can be interpreted as a meaningful sequence. In the case of clustering, the aim is to find clusters, but seriation has a more diffuse purpose of visual representation. For example, seriation helps to visually detect objects and variables with large amount of missing data and outliers. Seriation provides heatmaps with reasonable less striped features. The results are quite often arrangements around the diagonal or visually detectable clusters. Seriation can be performed not only on measured data, but on, e.g., model parameters, neuron intensities in artificial neural networks, connection data, as well. In these cases, seriation might help in the interpretation of the models and the operations.

Liiv started to unify the taxonomy of the different methods [5]. Theoretically, seriation means the permutation of data stored in one-dimensional vectors up to *k*-dimensional arrays. There are modes and ways in seriation. A mode means an independent sequence that can be permutated. Way is the dimensionality of the object used in visual perception, during the calculation of a merit function, or during a prescribed set of operations. The number of modes and ways mostly coincide to the dimension of the data. In chemistry, we often have two-dimensional data matrices with *N* rows connected to the objects and *M* variables denoting the columns. When we sequence both the objects and the variables in a classical data table, we perform two-mode–two-way seriation. When we sequence only the objects, we usually calculate a symmetric *NxN* distance matrix, and the seriation is one-mode–two-way. If we seriate only the variable sequence, the *MxM* covariance matrix might be a reasonable choice and the seriation is one-mode–two-way.

There is rather large number of seriation methods. There is not any canonical way, the popular methods differ from field to field. Large number of applications can be found on the field of bioinformatics, where, e.g., the node deleting algorithm of biclustering is one of the most popular methods [15]. There are two groups of the methods. In a part of them, a mathematical merit (or loss) function is defined which depends on the sequencing of the modes. In the other cases, set of operational instructions are used. In the first case, the extremum of the merit or loss function can be found by any global optimization scheme, e.g., simulated annealing, genetic algorithm or other specialized solutions. These methods are mostly iterative, and they use a stop criterion. An example of the operational methods is the barycentric heuristic algorithm. Here, ranks are calculated at the given state of the matrix for each rows/columns and the matrix rows and columns are sorted according to these ranks. Thereafter, the ranks are recalculated, and the matrix is sorted again. [9, 22]. The operational instructions are repeated as long as the condition of the operation is holding. There might be some extra conditions to avoid infinite loops and it is worthwhile to start all methods from several sequences whereof many can be randomized ones. For operational algorithms the schematic presentation of the problem on graphs can be useful [9, 22]. A part of the algorithms was inspired by the minimal number of crossings known as Turán’s brick factory problem in mathematics [23].

Another aspect of the two-mode seriations is whether the two sequences are treated independent from each other, or the merit function/operation contain cross terms. For the independent case, an example is the seriation of the objects according to the distance matrix and seriation of the variables according to the covariance matrix. Despite the independence of the two modes, by chance, we might get clearly interpretable data, where relations between the two axes are easily readable, e.g., on heatmaps. For having dependent two-mode seriation, we need cross terms between the sequences or geometrical preferences of the matrices. A recipe is sometimes that we have several local function values and the sum or the spatially weighted sum of the local functions provide the merit of loss function. The local functions might relate simply to the increase, to the decrease, or to the modality of the data within row or column wise and, e.g., the global loss function is the number of the violated case. For an overview of some of the methods and mathematical details, we refer to the review of Liiv [5] and the study of Hahsler et al. [11]. A few traditional merit/loss functions are described in the Additional file 1 in order to help the comparison to our method.

In our previous studies [7, 21] a local feature was calculated as the distance of two objects in a limited variable-vector space. We used three-variable spaces and the *i-j* element of the so-called local distance matrix contained the average distance of the *i*-th object to its sequential neighbours in the local space formed by the *j-1,j*, and *j + 1* variables. The global merit function was a weighted sum of these local distances, where the weights were the spatial distance of the *i-j* matrix elements from the diagonal of the data matrix. The algorithm provided that low local distances were sequenced around the diagonal of the data matrix. The ordering was according to one visual feature, it ordered similar objects close to each other and the corresponding variables around the corresponding diagonal parts were suggested to be responsible for the similarity.

We experienced that only a part of chemical data is meaningful in the obtained block diagonal forms. For example, there might be a group of variables responsible for two or more clusters of objects that is not easy to detect visually on a narrow diagonal-like arrangement. Our first idea was to improve our previous method by introduction of further adaptive lines with similar task as the diagonal had, but during the elaboration we realized that it is easier to think on a seriation forming patches.

In this paper we show our new method where the local function is a local average similarity, and the global merit function is the sum of the products of the neighbouring local similarities. We found that this merit function forms patches of the neighbouring objects and variables. A patch means a local space, where the given objects are similar to each other. In our philosophy, the object-variable points outside the patches are not relevant for the similarity patterns. We show it on simple chemical data, as well as on model details of artificial neural networks (ANN). The latter is related to the interpretation [24] of ANN models, e.g., we were able to interpret the roles of the neurons connected to the variables and to the objects. The former means the seriation of the weights in the network and the latter was managed by seriation of activities caused the different objects on the different neurons.

Our method can be easily extended to higher modes and ways seriations. We developed different three-mode-three-way methods. Since it is less easy to interpret three-dimensional data structures than two-dimensional ones, we usually used projections onto two-dimensional heatmaps, here. A part of our results is shown on co-plots elaborated by us, where the original data heatmap and local similarity contour plot are merged. It helps to easily find the responsible variables for the similarity of a cluster of variables.

## Theory

In 2011 we introduced a mathematical merit function for 1-mode-2-way and 2-mode-2-way seriation of matrices [7]. Two concepts were introduced there. The local distance matrix contained the average distance between the *i*-th object and its two neighbours in a local three-variable space, where the index of the middle variable assigned to *j*. The other quantity we called diagonal measure, and it represented the distribution of the elements of the local distance. It was the scalar sum of the local distances weighted with their positional distance from the diagonal of the matrix. In 2-mode-2-way seriation the diagonal measure was maximized to order similar objects close to each other and the corresponding variables around the diagonal were suggested to be responsible for the similarity. If distance matrix of the objects was 1-mode-2 way seriated, the diagonal measure was maximized, as well. If covariance matrix of variables was 1-mode-2-way seriated, the diagonal measure was minimized. In our new research we propose the development of our idea both on the local quantity and the global measure.

### Local similarity matrix

Distances are unbounded positive numbers, what may hinder the interpretation of the actual values. Algorithmically, it is more convenient to use bounded set of values. Similarity is a frequently used concept for that. There is a reciprocal relation between similarity, a value of one denotes perfect similarity of two objects (zero distances of the objects in the variable space) and zero similarity means maximal distance between the objects. There are different definitions of similarity, whereof we finally selected that *similarity = 1-(distance/maximal distance)* equation. We defined a local similarity matrix (S) similarly to the local distance matrix. *s*_*ij*_ shows how the *i*-th object is similar to its neighbours in a local 3-variable space around variable *j*. For 2-mode-2way seriation it is calculated as:

1 $$l_{{i,k,j}} \, = \,~\sqrt {\sum\nolimits_{{l = j - 1}}^{{j + 1}} {\left( {\frac{{a_{{kl}} \, - \,a_{{il}} }}{{diff\,_{{max,l}} }}} \right)^{2} } }$$

2 $$s_{{ij}} \, = \,\left( {\sum\nolimits_{{k = i - 1,i + 1}}^{{}} {1\, - ~\,\frac{{l_{{i,k,j}} }}{{D_{{col,j}} }}} } \right)/D_{{row,i}}$$

,where *a*_*il*_ and *a*_*kl*_ are the elements of the *A* matrix to be seriated, *l*_*ikj*_ is their local distance in the variable space formed by the *l = j-1, j* and *j + 1* variables. *k* takes only the values *i-1* and *i + 1* for a given *i* index of objects. *diff*_max,l_ is the difference between the largest and the smallest elements of the *l*-th column in *A*. It is used to scale the distance between [0,sqrt(3)], if the local variable space contains three variables. If the *j*-th variable is at the first or the last column of the matrix, the local space contains only [0,sqrt(2)]scaled distances. *D*_*col,j*_ contains the corresponding upper bounds of the intervals for each variable. *D*_*row,I*_ is usually two for the *i*-th object except the first and the last row, where it is one. These row or column dependent quantities (*diff*_*maxl*_, *D*_*col,j*_, *D*_*row,i*_) were introduced to be able to get theoretically *s*_ij_ ϵ [0,1] values for all *i-j* positions including the non-bulk matrix elements.

### The global patch function

In the case of our previous global scalar (diagonal measure), the seriated matrix placed the variables responsible for object similarities around the corresponding part of the diagonal. It means, only the most important variables were emphasized, and, e.g., there was no possibility to select a variable to be important for several object clusters. In our new method we define a merit function, where forming of several patches is supported by maximising it in 2-mode-2way seriation. If we calculate the sum of the product of two neighbouring local similarity values (*P*), this quantity reflects the spatial distribution of large and small similarities. If random order of objects and variables is used, the local similarities are distributed randomly in the matrix. If we seriate the matrix to have larger sum of neighbouring products, a higher sum can be reached by clustering high and low local similarities separately. Furthermore, preferential rearrangement is also supported by maximising such a merit function which creates higher similarities by neighbouring similar objects. In the high similarity patches the objects are similar in the local variable space and both the objects and the variables can be identified.

3 $$P\, = \,\sum\nolimits_{{i = 1}}^{{n - 1}} {\sum\nolimits_{{j = 1}}^{m} {2\left( {s_{{ij}} s_{{i + 1,j}} } \right)^{q} } } \, + \,\sum\nolimits_{{i = 1}}^{n} {\sum\nolimits_{{j = 1}}^{{m - 1}} {2\left( {s_{{ij}} s_{{i,j + 1}} } \right)^{q} } } ~$$

, where *i-*s are the row and *j-*s are the column indices of the *nXm* local similarity matrix and *q* is an arbitrary contrast. The two effects of maximising *P* - spatial ordering and creation of high similarities - can be justified separately. The simple rearrangement of any matrix by clustering large and small values provides large *P*: it is similar to a negative local entropy. We performed several test calculations supported this, and there is also a thought experiment in the supporting material. The other effect is straightforward, that placing similar objects and variables close to each other increases the sum of the local similarities. The exponent *q* is an empirical contrast factor. At high *q* values the positioning of highest similarities close to each other is extremely preferential and it may cause compact and small clusters, while small *q*-s do not penalize so strictly the less large values, it may cause slightly larger patches. We used *q* = 2 and *q* = 3 in our calculation. Depending on the dataset, the visual results was sometimes better for one of the *q* choices, but it did not seem to be a decisive parameter of the merit function. We note, that our patch function was obtained after several trials, where at first, we focused on entropy or Gini-index like approximations. We found, that *P* defined as in Eq. 3 is a simple and feasible merit function.

If we would like to highlight the pros and cons of our merit function, we might compare it to the features of existing ones (see some details in [11] and in the Additional file 1). In the case of two-mode-two-way seriations, the most functions do not connect the two modes. The modes are seriated independently and there is only a chance that the sequences of the two modes have some cross relevance. The Moore-stress [25] is an exception (see Additional file 1), but there, the roles of the rows and columns are identical. It means, there are no differences in the object and variable spaces. In our case, only the variable space is used to define distances/similarities/dissimilarities that is closer to the basic features of data matrices. In the same time our local similarity matrix connects the two modes, similarly to the Moore-stress. In the case of other merit functions, most of them are simple sums of values (e.g., Moore and Neumann stresses [25]) without any link to spatial arrangements or they reflect only spatial arrangements (e.g., violation of Robinsonian trends [26]). Our patch function is unique by taking care both on maximising the local similarity elements and spatially arranging them. This was valid also for our previous diagonal measure/local distance matrix scheme, but the present patch function/local similarity scheme is more flexible. The last advantage of our method is the local feature, namely that only the locally important variables are used in the calculation in contrary to the most clustering methods. It means, a local smoothness is forced instead of putting rows together with similarity everywhere. The disadvantage of our method is that up to now we have not found a computationally cheap method to optimize our merit function.

### Local similarities in higher dimensions

The generalization of the local similarity matrix and the patch function to higher dimensions can be easily done, if we follow the idea that we are interested in the average similarity of an object to its sequential neighbours in a local three-variable space. The calculation of the possible cases, e.g., the dimension of the original data, the dimension of the local similarity matrix, the number of possible local variable sets are detailed in the Results and Discussion section together with some examples. The corresponding equations for the cases are shown in the Additional file 1. We show three possibilities for three-dimensional local similarities, where the three axes are formed by one object and two variable vectors (OVV case, original data are 2D), by two object and one variable vectors (OOV-independent, the original data are two dimensional) and by another two object and one variable vectors case (OOV-dependent, the original data are three dimensional).

### Missing data and noninformative zeros

There are several datasets in chemistry, where part of the data is missing. The causes might be different, e.g., lack of general experimental methods for all objects, operational break down, or the given variable is not relevant for that object. In the case of cheminformatics, it is also common, that several extra variables are added to the database where most of the objects provides a zero value. An example is the presence of chemical groups, if close to all the molecules do not contain that functional group. The traditional method to overwrite the missing data with an average or random value might bias the seriation. Therefore, it would be feasible to avoid the replacement of missing data. Also, it is rather misleading, if the unnecessary and irrelevant zeros have crucial effect on the merit function of seriation. We solve the problem of missing data and unnecessary zeros by proposing a different calculation of the local similarities for these cases:

4 $$s_{{ij}} \, = \,\sum\nolimits_{{k = i - 1,i + 1}}^{{}} {\sum\nolimits_{{l = j - 1}}^{{j + 1}} {\left( {1 - \left| {\frac{{a_{{kl}} - a_{{il}} }}{{diff\,_{{max,l}} }}} \right|} \right)/6} } ~$$

The inner sum is skipped for all data, where any of the data (*a*_*kl*_ or *a*_*il*_) is non-existent. It can be used for missing data as well as for unnecessary zero values. Using Eq.  4 the local similarity cannot be one, if there are undetermined cases in the sum. Also, if *s*_*ij*_ refers to a matrix position at edges or corners, the possibility for the local similarities to be 1 is excluded, there maximum value is 2/3, 1/2, or 1/3. This handling of the borders is different from Eq.  2. The patch function is calculated according to Eq.  3. There is only one difference, there might be a chance that a *s*_ij_ remains undetermined. In that case the undetermined *s*_*ij*_ is skipped in Eq. 3.

Equations  1– 2 provide a local average similarity calculated on distances of L2 norm in a three-dimensional local variable space (average of two local similarities where each of them was calculated using three-dimensional distances). On contrary, Eq.  4 calculates the similarities independently for each local variables and sum the 1/6 of these similarities (it might be interpreted as 1-L1norm(first object pair)/3-L1norm(second object pair)/3.). Both methods are accessible in our code for data matrices without missing data or zeros to be omitted. If missing data or unnecessary zeros are present, only Eq. 4 is accessible in the code. We were not able to find any theoretical reasoning why Eqs.  1– 2. (modified L2 norm to have theoretically *s*_ij_ ϵ [0,1] everywhere) or Eq.  4 (using L1 norm without correction at edges) is theoretically feasible. Therefore, we leave both options open for the users.

## Calculation details

### Codes

The patch seriation was performed using a C code developed in our laboratory. The code reads the datasets, manages data pre-processing as optional normalization, scaling, changing zeros to undetermined values. The maximization of the patch function was obtained with Metropolis Monte Carlo algorithm, where the ordering with the largest *P* was stored as the best one. The acceptance ratios for the different type of trial changes were set to be around 0.05. Column and row permutations were performed independently. In the case of three-mode-three-way seriation it was performed independently for all modes. The number of the trials was 1–5 million. A calculation took a few minutes on a PC depending on the size of the dataset. During this calculation length, usually the best sequence was detected and stored at any time after the 20% of the calculation time. A few (2–5) seriations were performed for each dataset at *q = 2* and *q = 3* values, which of the results to be shown were selected visually.

Schematically the calculation starts with a matrix with randomized row and column sequences. Thereafter exchange of two rows or columns are performed. The step is accepted, if the merit function of the new arrangement is larger than that of the old one. The global optimization is maintained by Neumann’s rejection method, small decrease of the merit function is also accepted with a small probability to have a total acceptance ratio of 0.05. In a few cases during the calculation, the ’temperature’ of the Metropolis algorithm is increased temporary to restart from new arrangements. The sequence with the largest merit function is stored as the final result.

The elaboration and visualization of seriation results were done using R [12]. In the comparison to other methods, here we used the seriation package of Hahsler et al. [11]. For three-dimensional graphs we used the RGL package [27]. We developed an overlay plot, where the heatmap coded scaled data are shown together with contour plots of the local similarity. We think, these overlay plots are rather effective to identify object clusters and the variables causing the similarity.

The neural network modelling was performed in Python using the scikit learn package [13]. The partly optimized hyperparameter sets for the models were selected from one of our previous studies where we used the same datasets [28].

### Datasets

The tested datasets are mostly freely available ones related to QSAR, chemistry, material science, food science, cheminformatics and environmental chemistry. Several datasets of them are accessible in repositories [29–31]. Some details of the data and the performed type of seriations are collected in Table 1. The first dataset (SIM [32]) is a semi-randomly simulated one, its structure is related to our initial idea, what kind of benefit we would like to get using patch seriation. There are 50 objects and 20 variables in this set ordered in 4 clusters of 10 objects each and 10 random objects not associated with any clusters. Members of the clusters have similar values at some selected variables, but their other data are random. Some of the selected variables are common also with other clusters. At first, we generated [0,1) random numbers for all data and thereafter the groups were recalculated by adding a given random number for that variable of the group biased with white noise. In the case of other datasets, if a dataset was published for modelling a response variable, we omitted it from the seriation and only the predictor variables were used in the seriation process.

The RETSIM dataset [32] is a simulated one, as well. We defined three functional groups and created 4 compounds with random linear combination of the three groups. We set 6 mixtures of the 4 compounds. 6 chromatographic columns were set as well with differently randomized partial retention times for the functional groups. The retention times of the compounds were calculated with linear combination of the functional groups therein. Finally, we added uniform broadening for each compound with integrals related to the concentrations. In this way we had 36 chromatograms of the 6 mixtures on the 6 columns.

**Table 1:** Datasets

Abbr.	Row × column	Description	Seriations	Refs.
SIM	50 × 20	4 clusters with common variables for each	OV	[32]
RETSIM	(6 × 6)x100 and (6 × 6)x150	Simulated retention times of mixtures on different columns (+’fingerprints’)	OV; OOV dependent	[32]
POL_MONTH	(26 × 12)x9	Monthly air pollutant averages at 26 stations in 2017	OV; OOV dependent	[33]
POL_YEAR	(12 + 14)x9	Yearly air pollutant averages at 26 stations in 2017 (12 at Budapest, 14 at countryside)	OV; OOV independent	[33]
FLASHP1	420 × 26 for ANN model (N = 4,6,8,10 hidden neurons), 80 objects in the test set	Flash point estimation of molecules using different QSAR parameters	OV: 80 × 26 (test objects-variables); 26xN (variables neuron weights); Nx80 (neurons, object activities on the neurons)	[29, 34]
DR8	600 × 28 for ANN model (N = 10–15 neurons), 114 objects in the test set	Different QSAR parameters originally used to estimate toxicity	OV: 114 × 28 (test objects-variables); 28xN (variables-neuron weights); Nx114 (neurons-object activities on the neurons); OVV independent 114x(N + 28) (objects, neuron activities, original variables)	[29, 35]
FLASHP2	632 × (13 + 12), for ANN models (N = 10–15 neurons), 100 object in the test set	13 molecular and 12 general descriptors of molecules originally used for flash point estimation	OV of 100 × 25, 100 × 13, 100 × 12; OVV 100 × (13 + 12); OVV 114x(N + 28) (objects, neuron activities, original variables)	[29, 36]
POLMET_DAY	56 × (7 + 6), subset of original, two weeks from each season (56 days)	Daily meterological and airpollutant data set in 2007	OV: 56 × 13, 56 × 7, 56 × 6; 3D OVV: 56 × (7 + 6)	[37]
ESSOIL	10 × (10 + 38)	Essential oils in 10 species, 10 chemical and 38 bactericid/fungicide data	OV: 10 × 48, 48 × 10; 3D OVV: 10 × (10 + 38)	[8]
CERAMIC	88 × 17	Ceramics with body and glaze data	OV	[31, 38]
GLASS	214 × 9	Composition of glasses from different sources	OV	[30, 31, 39]
WINE	178 × 13	Wine analysis	OV	[30, 40]
TOXIC	112 × 8	Toxic on 8 data	OV	[41]
SAND	30 × 14	Sand data radiation	OV	[42]
MOLDESCRg	500 × 50 more subsets of the original	Molecular descriptors for enormous number of molecules to calculate different properties	OV	[29, 43]
COIN	257 × 10	Composition of ancient coins from different era of Hungary	OV	[44–46]
REAC	95 × 32	Fuel combustion with reactions and reactants	OV	[32, 47]

## Results and discussion

### Simulated dataset for 2-mode-2-way seriation

**Fig. 1:**
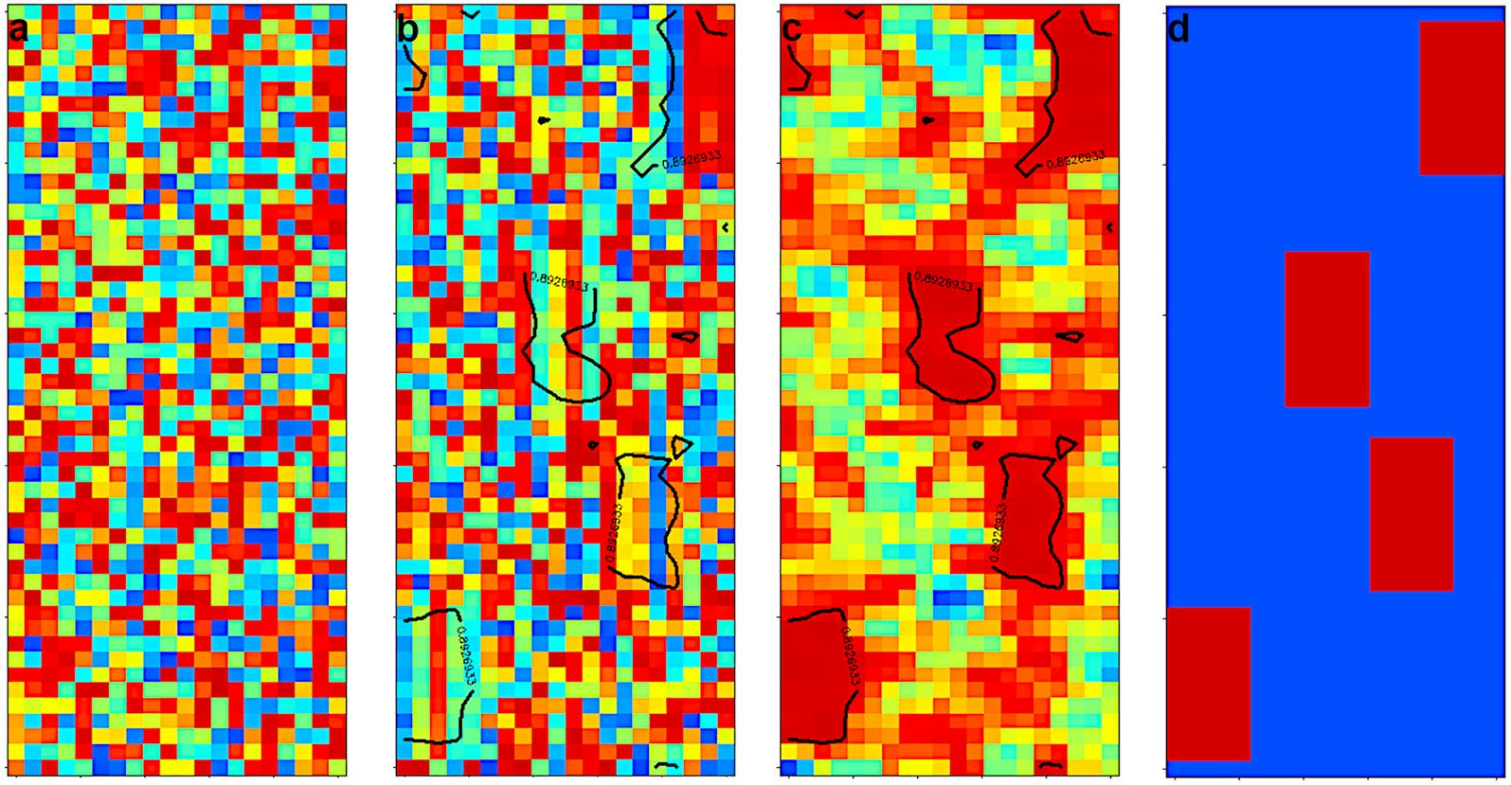
2-mode-2-way seriation of simulated data. The data are shown on scaled heatmaps. **a** random order (typical start) **b** an example of patch seriated data with *q* = 3. The overlay contour plot shows the local similarity levels around 0.85. **c** the corresponding local similarity matrix sorted as figure **b**, **d** intended common values of the clusters during the dataset simulation sorted as figure **b**

The dataset contained 50 objects and 20 variables. Each of the 4 clusters had 10 objects. The objects within a cluster had similar values in the case of 5 variables and they had random ones for the other 15 variables. These variables were distinct except for group C and D, here two variables were common but with different average values for the two sets of objects. Ten objects and two variables had no cluster affiliation. Figure 1 shows the heatmaps of a randomly ordered matrix (used as start), the seriated data matrix, the corresponding local similarity matrix and the corresponding hidden cluster information at the data generation in the seriated order. If we plot only the data matrix as a heatmap, it is not easy to identify the clusters. Therefore, we use the overlay of a contour plot on the local similarity matrix in Fig. 1b. In more than one third of our trials the variables were seriated perfectly and the number of clusters of the objects was equal or only slightly more than 4. We show a case in Fig. 1b and d, where the seriation worked perfectly both for variables and objects. We choose the actual contour levels of the local similarity matrix to help the assignment of the clusters in Fig. 1b. The 10 random objects and the 2 random variables are sometimes between the clusters or sometimes they are neighbouring to each other, but the overlay contour plot does not identify them as a 5th cluster.

The number of the object clusters is 4 in this example. We compared it to other seriation methods built in R [11, 12]. The number of object clusters were between 14 and 33 for the other methods. It means, the clusters were split into 3–8 parts in average. We also calculated how many of the variables are found in a cluster for the object clusters. In our seriations, the 5 variables were mostly clustered for all object groups correctly. In the case of the other methods, it was between 13 and 18. It means, there were only 2–7 cases, when two common variables of object clusters were placed to be neighbours in the variable sequence. The Additional file 1 contains further details on the comparison of the methods. We should emphasize, that our patch seriation worked efficiently both for objects and variables simultaneously and it explores the link between the two modes. This link is missing for most of the other methods. As it can be seen in the Additional file 1, most of the methods use only distance matrices of the objects, where the simultaneous sequencing of the two axes is not possible. In our comparison, we calculated also variable sequencing of the other methods by calculating a ‘distance matrix’ of the variables, as well.

In this prototype of data, different groups of variables are responsible for the different clusters of the objects and the other variables are not important for the object clustering. It seems so, that our method totally outperforms all the other seriation methods. Even more, clustering methods, e.g., hierarchical clustering is not able to find this type of relationship within the objects due to the nonlocal distance calculations.

### 2-mode-2-way seriation of other datasets

**Fig. 2:**
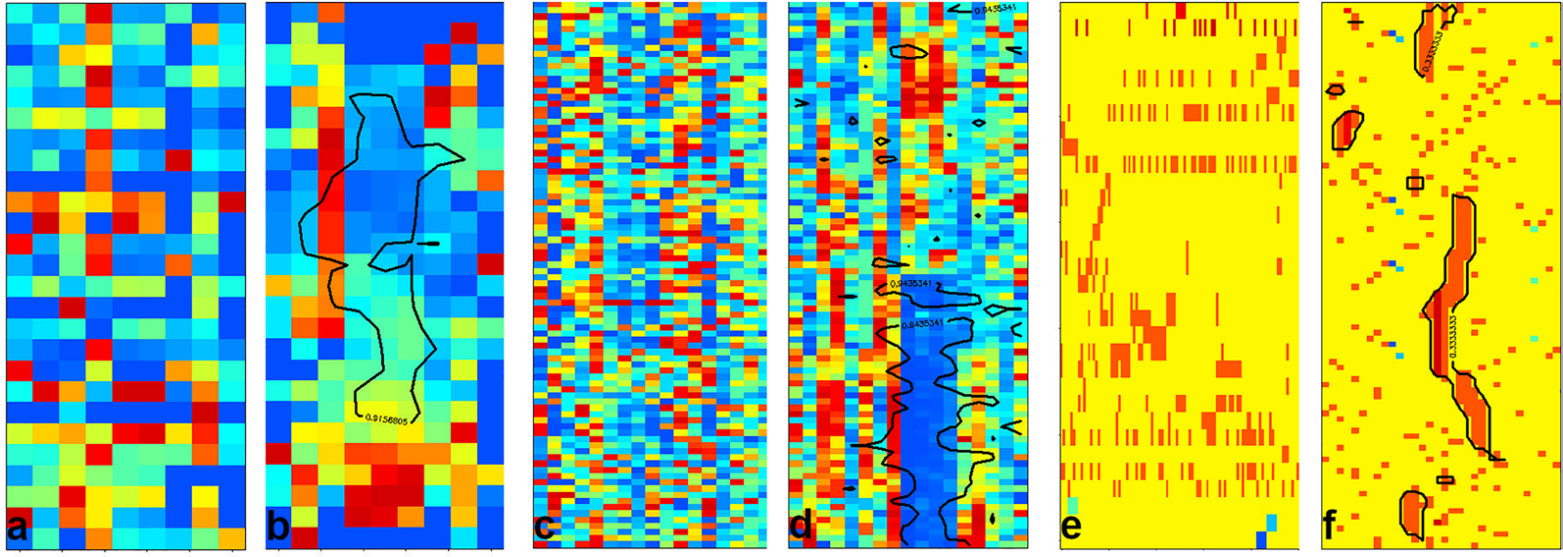
2-mode-2-way seriation. **a**, **c**, **e**: random data order **b**, **d**, **f**: seriated ones. The data are shown on scaled heatmaps. **a**, **b** yearly air pollution data at 26 stations in 2017 (POL_YEAR dataset), the seriated order of the variables (columns): PM2.5, PM10, O_3_, NO_2_, NO_X_, NO, SO_2_, CO, BENZOL **c**–**d**) components of celadon ceramics (CERAMIC dataset) **e**–**f** reaction and species in a gasoline combustion model (REAC dataset), blue denotes the three reactants, red ones are H_2_O and CO_2_

The most of the tested patch seriations concerned the simultaneous ordering of objects and variables in two dimensions. Here we show some examples with original and seriated heatmaps. Figure 2. shows three datasets.

The first is the POL_YEAR one [33] in Fig. 2a–b. It contains the yearly averaged concentrations of 9 air pollutants at 12 places in Budapest and 14 places at countryside (mostly in cities) in 2017. There are differences in the measuring stations, not all of them were able to measure all the 9 components and there were several shutdowns at some places. Here, we calculated the local similarity matrix according to Eq. 4. The seriation clearly shows that the dominant variables for similarity are the different nitrogen-oxide and ozone concentrations. The stations with heavy traffic are ordered close to each other. The other stations are separated into two groups, where the nitrogen oxides are dominant pollutants and where ozone pollution is dominant. It is known, that there is a transition cycle of ozone and nitrogen-dioxide. The first and the last stations are somehow thrown out by the seriation. These are the places where the number of missing data was high.

In Fig. 2c–d we show a case, where the component of ceramics (celadons) are the variables [38]. Here it is known for the objects, what kind of celadon ceramics and what part of them are analysed (body or glaze). The seriation clearly shows, how the randomized data can be turned to form two groups: body and glaze, according to the low concentration of some oxides in the body part and high concentrations of them in the glaze. The seriation also found a subgroup of celadons, which were only imitated Longquan celadon in Jingdezhen civilian kilns in Ming Dynasty. The seriation was not able to differentiate the Longquan celadons of different dynasties.

In Fig. 2e–f we show a set of reactions and reactants used in the combustion modelling of gasoline [47]. It is not easy to determine an order of the reactions and reactants. Previously we used our diagonal seriation for this reaction set and we were able to arrange them according to a diagonal suggesting a hypothetical pathway. Of course, that simple order was related to a non-realistic graph structure, where it is well known that a proposed way need not coincidence to the real fluxes of the processes, especially the fluxes highly differ for different combustion conditions. In the case of patch seriation, we concentrated on the identification of reaction parts, e.g., reactions using the same components as reactants or products. The presence of a component in a reaction was denoted with 1 irrespectively the components role and stoichiometry. The gasoline components, the final CO_2_ and H_2_O components are coloured differently. It can be seen in Fig. 2f, that there is a reasonable re-clustering of the reaction system, where around six clusters of reaction-components are there. Two of them is related to the final products CO_2_ and H_2_O, another is related to the CH_4_ and CH_3_ components, one is formed by different small entities containing H and O, and another contains additionally carbons. The group on the bottom-middle is related to H and H_2_. Such kind of seriation might be interesting if one intends to build reaction mechanism in a modular way.

**Fig. 3:**
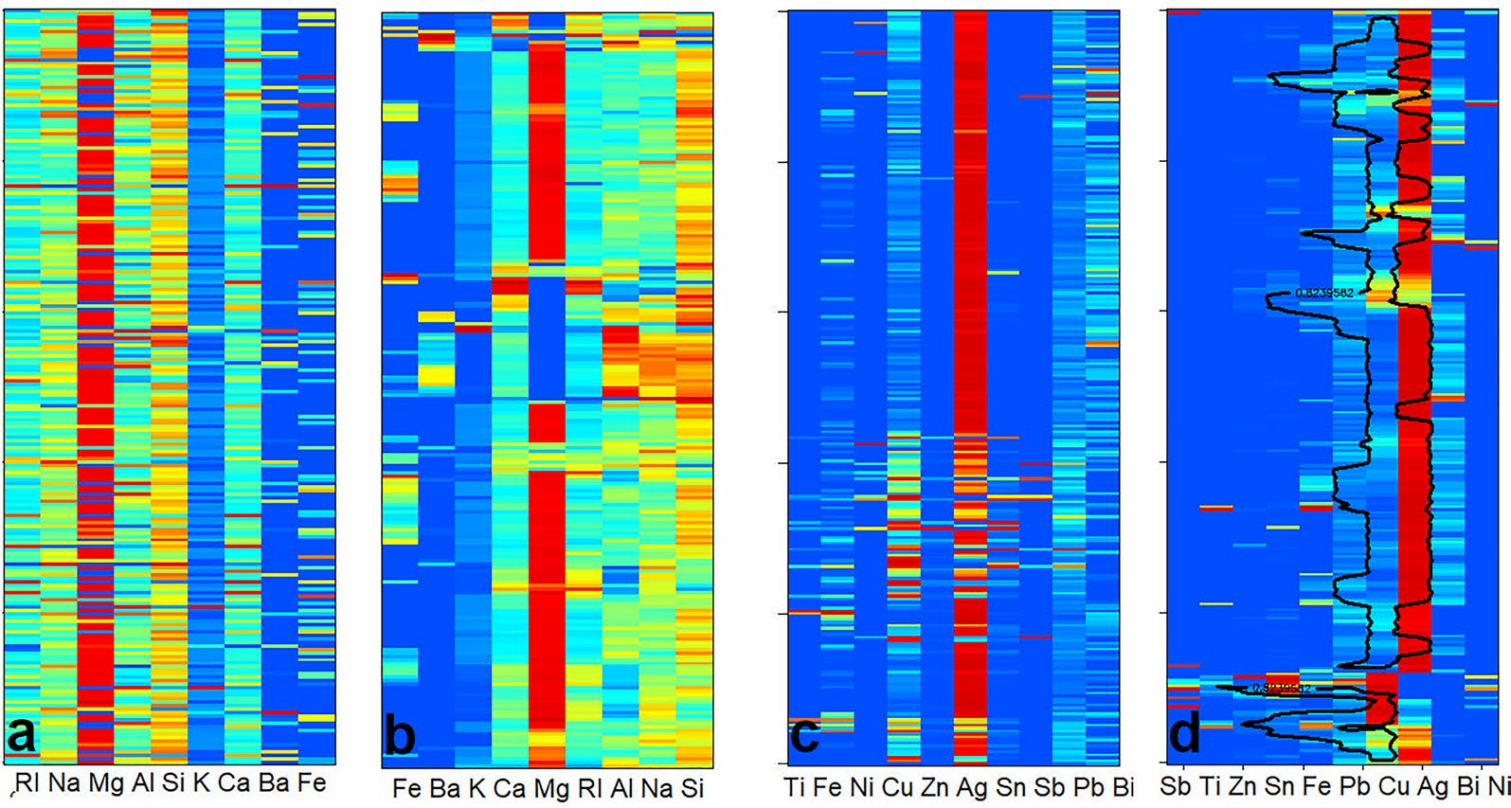
2-mode-2-way seriation. The data are shown on scaled heatmaps. **a**–**b** glass compositions (GLASS dataset) **a**-random, **b**-seriated **c–d** coin compositions (COIN) **c**-random, **d**-seriated

In Fig. 3 we show two cases, where the seriation helps to find some general pattern. In the GLASS and COIN compositional datasets the exchange of species can be easily detected in the seriated data besides the visual simplification of the heatmaps. In the seriated GLASS data (Fig. 3b) one can detect a negative mirror like difference in the exchange of ions with similar charges, e.g., K^+^ - Na^+^; Ca^2+^ - Mg^2+^; Al^3+^ - Ba^3+^. Furthermore, it is also easy to detect the relation between Al^3+^ and the refractive index. It is not easy to realize these features in the unseriated data according to the rather striped heatmap (Fig. 3a). The method also seriated the glasses quite well according to their sources or use detailed in the original source [30, 31, 39].

Figure 3c–d contain a dataset on Hungarian coins from the X-XIII. century. Here, we used Eq. 4 and set the zero values to be skipped during the calculation of the local similarity matrix. The heatmap shows a similar exchange of species, like Cu-Ag exchange. It groups the coins where Sn and Sb took part in it, as well. The seriation was done with 0–1 scaled data, therefore, the traces of some metals had a large effect on the ordering. Due to the scaling, it is easy to identify the coins having the same metals from second importance. Our method clusters the objects (coins) according to the era and kings, but here we should add that traditional clustering and classification methods provided better results [44, 45].

### 3D seriation: 1-object–2-variables case

If we have a two-dimensional data matrix, where the columns contain two separable set of variables, we might perform a seriation, where the order of the objects, the first set variables and the second set of variables can be separately sequenced. A three-dimensional local similarity matrix can be constructed, where an element shows the average similarity of the given object to its neighbours (axis one), but now in two local three-dimensional variable spaces (axis two or axis three). Using the first type of variables and the second type of variables independently, we calculate the two local similarities between the given object and one of its neighbours. The final *s*_ijk_ local similarity contains the average of four similarities (over the two neighbours times the two local variable spaces).

Such an independent set of variables can be, e.g., the chemical content and the biological activities of the essential oils (ESSOIL), the daily averages of air pollutants and the corresponding meteorological data (POLMET-DAY) or the complex QSAR descriptors and the simple enumeration of functional groups in the flash point modelling FLASHP2.

**Fig. 4:**
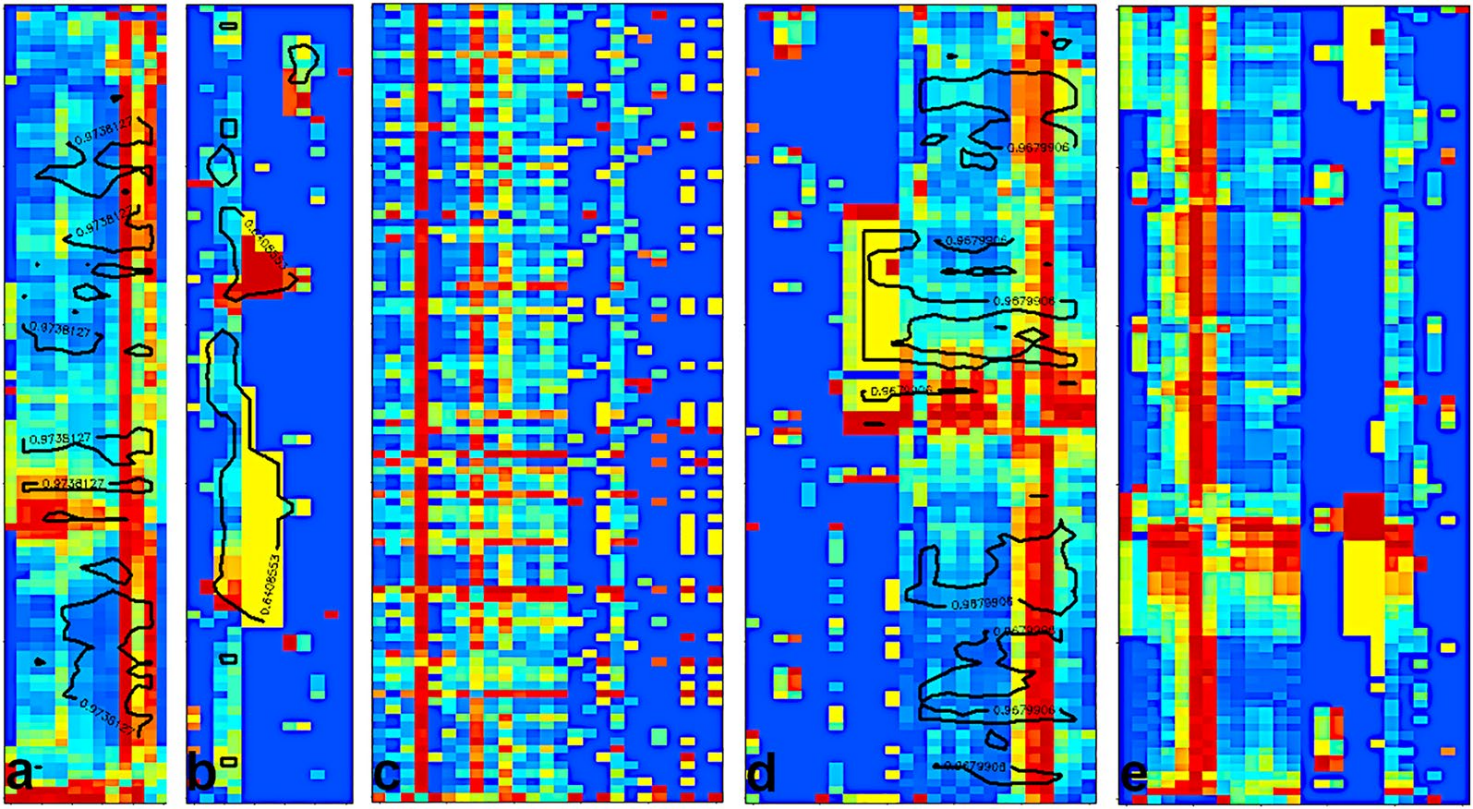
2-mode-2-way seriation and 3-mode-3-way seriation of the FLASHP2 dataset. **a** 2-mode-2-way seriation of the objects with molecular descriptors **b** 2-mode-2-way seriation of the objects with general descriptors **c** Random start of the whole data matrix used in 2-mode-2-way seriation **d** 2-mode-2-way seriated whole data matrix **e** 3-mode-3-way seriated whole data matrix unfolded to 2D

**Fig. 5:**
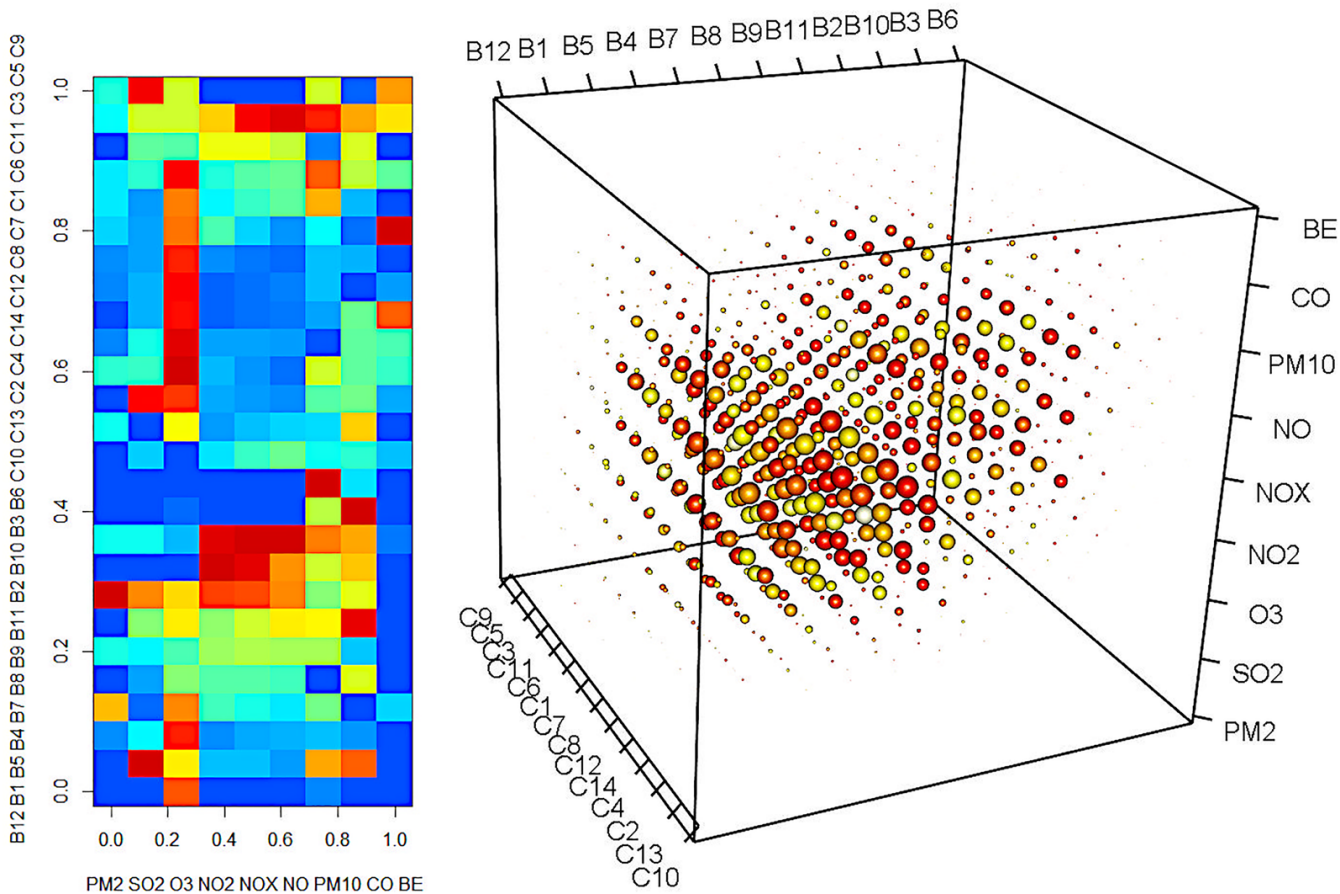
3-mode-3-way seriation of the POL-YEAR dataset. Right: seriated data matrix (stations in Budapest and at countryside form the two independent object sets. Left: three-dimensional view of the local similarity array. B1-B12: stations at Budapest in alphabetical order, C1-C14: stations at countryside in alphabetical order, PM2 = PM2.5, BE = benzene

We show our results on the latter one, where the QSAR descriptors for a given molecule are called molecular descriptors in the original paper and the enumeration of functional groups is called general descriptors. If we apply separately two-dimensional seriation for the two sets of variables, the obtained heatmaps are clearly arranged (Fig. 4a–b). Here we calculated the local similarities with skipping the zero data to avoid the clustering of molecules due to the lack of functional groups in the set. If we seriated the total data matrix in two-dimensions, many of the clear patches disappeared. The continuous molecular variables were dominant during the seriation, most of the general descriptors was not clearly seriated (Fig. 4d). If we performed the seriation using a three-dimensional local similarity matrix, the two parts of the data in the original two-dimensional dataset provided clear patches for both set of variables (projected back to two dimensions: Fig. 4e). The advantage of the three-dimensional seriation over the two independent two-dimensional seriations is the common target function during the sequencing of the three axes. The three-dimensional local similarity array can be directly visualized (see later Fig. 5 right) or two-dimensional projections can be calculated (see Additional file 1). We emphasize here again, that our mathematical target function connects all the modes of the seriation in contrary to the usual biclustering schemes.

### 3D seriation: 2-objects-1-variable case

For the demonstration of the case, where a two-dimensional data matrix contains two different sets of objects, we selected the air pollution data of stations in Budapest and at countryside (POL-YEAR). The two sets of objects might be seriated independently. Here, the first axis of the local similarity matrix contains the stations at Budapest, the second one is the stations at countryside and the third axis shows the yearly averaged pollutant concentrations. The *s*_ijk_ local similarity contains the average of 4 similarities: the similarity of the *i-(i-1)* and *i-(i + 1)* object pairs of the first axis (stations in Budapest) and the *j-(j-1)* and *j-(j + 1)* object pairs of the second axis (stations at countryside). The local variable space for this element is spanned by the *k-1, k, k + 1* variables.

The results of the seriation can be shown in the original two-dimensional data (Fig. 5 right), where both set of stations are rather homogeneously sequenced separately (cf. to Fig. 2b. of 2D seriation, where the cities might be mixed). Using interactive three-dimensional graphics, one might have a look on the local similarity array. We show an example in Fig. 5 left, but it is rather uninformative without the possibility of rotating the graph. The different projections or enumerations on different subspaces of the three axes might be informative, e.g., which pollutant causes locations in Budapest and in countryside to be similar. It is clear from the graphs, that mostly the NO-NO_x_-NO_2_, and sometimes the SO_2_ and PM10 data cause the similarities. Table 2 shows this projection where highly similar locations are ordered into the middle of the local similarity array. The corresponding alphabetical code enumerates all the local variables involved in at a given high similarity, e.g., a similarity over 0.9 at the O_3_-NO_2_-NO_x_ position means that the two neighbouring variables (SO_2_ and NO) are also involved therein.

**Table 2:** Local similarities over 0.9 between stations in Budapest and in countryside

	1	2	3	4	5	6	7	8	9	10	11	12
1												
2			A	A	A	A						
3			A	A	B	B	C	D				
4			A	A	B	B	A					
5			A	A	B	E	A					
6			F	B	E	E	F	D				
7			F	E	E	E	G	D	D			
8			A	A	E	E	D	D				
9					D	H						
10			B	B	E	E	I					
11			A	A	A	A						
12												
13												
14												

The local variables causing the similarities: A: NO_2_,NO_X_,NO B: O_3_,NO_2_,NO_X_,NO C: NO_2_,NO_X_,NO,PM10 D: NO_X_,NO,PM10 E: SO_2_,O_3_,NO_2_,NO_X_,NO F: O_3_,NO_2_,NO_X_,NO,PM10 G: SO_2_,O_3_,NO_2_,NO_X_,NO,PM10 H: SO_2_,O_3_,NO_2_,NO_X_ I: O_3_,NO_2_,NO_X_ The stations belonging to the columns (Budapest) and the countryside (rows) are listed in the Additional file 1

### 3D seriation: 2-objects-1-variable sets – 3D original data

In the case of two dependent objects - one variable sets, the data might be originally three dimensional. In the case of the POL-MONTH dataset, the data are for 26 stations with 12 monthly averages of 9 pollutants in 2017. The theoretical three axes are stations, month and pollutants. The *s*_ijk_ local similarity contains the average of 4 similarities: the similarity of the *j-th* month data for the *i-(i-1)* and *i-(i + 1)* station column pairs and the similarity of the *i-th* station data for the *j-(j-1)* and *j-(j + 1)* monthly pairs. The local variable space for an element is spanned by the *k-1, k, k + 1* pollutant concentrations.

**Fig. 6:**
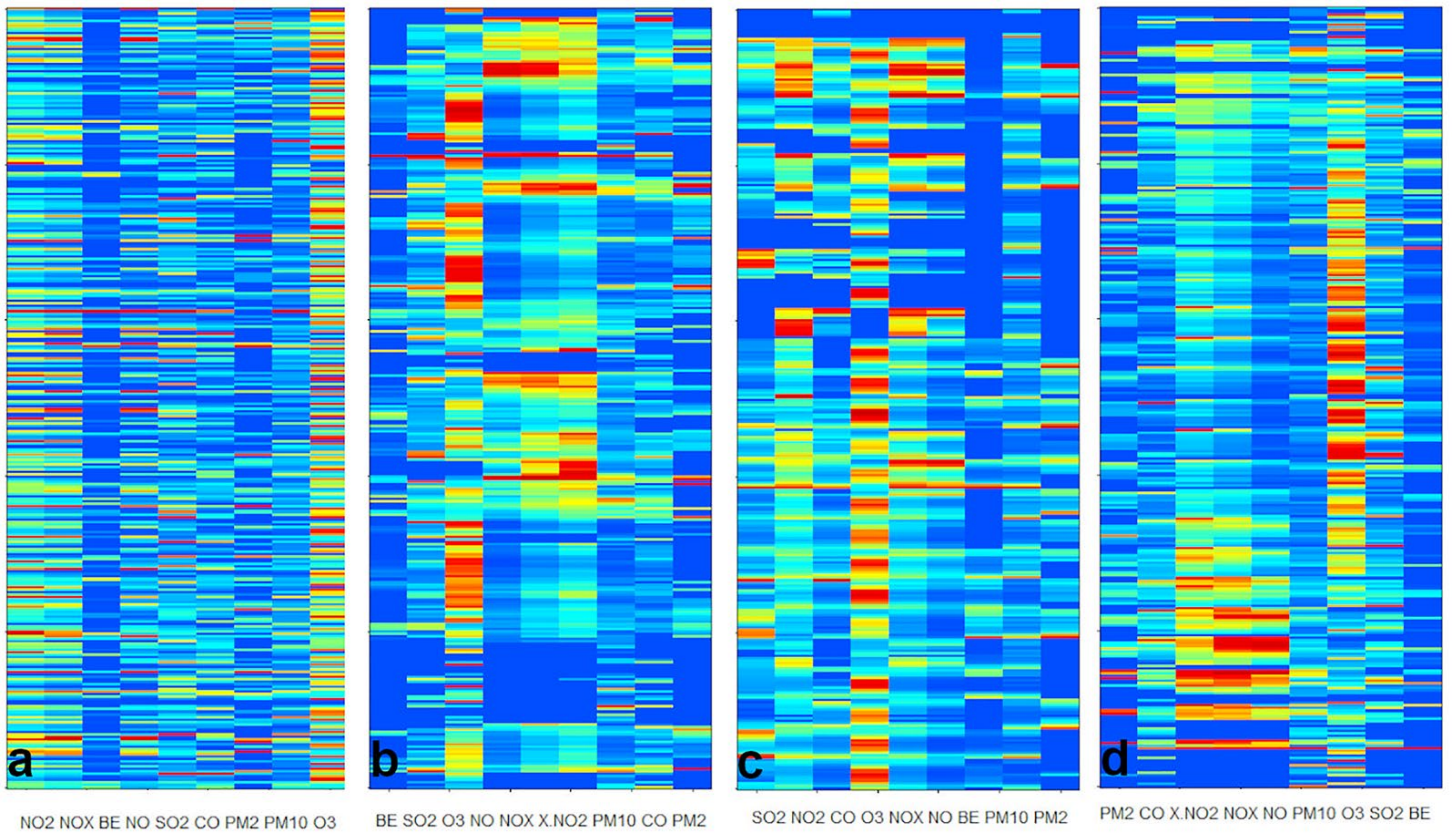
Seriation of monthly air pollutant averages at 26 stations in 2017 POL-MONTH. **a**–**b** 2-mode-2-way seriation **a** - random **b** - seriated. **c**, **d** 3-mode-3-way seriation **c** - ordered by hand, 12 months/station **d** - seriated

Figure 6a is a randomized data matrix where both rows (stations in a given month) and columns (pollutants) are randomized. If we perform a 2-mode-2-way patch seriation, the heatmap became simpler, e.g., the NO_2_, NO and NO_x_ variables were seriated near to each other (Fig. 6b). Here we used that zero and missing values were not used in the similarity calculations (Eq. 4). The stations and pollutants with a lot of missing values are out-seriated to the edges of the heatmap. One can see, as in the case of the monthly averages, that the high nitrogen-oxide and ozone data provided a good basis for similarity. Figure 6c shows the original data, where an arbitrary alphabetical order was used for the stations while the months are in calendar order. If we perform the 3-mode-3-way patch seriation, we obtained an ordered map with regular stripes (Fig. 6d). The neighbour analysis showed, that 48–59% of the neighbouring objects in the local similarity array belong to the same season, while this is only 39–47% for the 2-mode-2-way seriation. Around 30% of the four neighbours in the similarity array are the same in the station and/or in the month. We note that we do not want to get a perfect classification for these data, because it is not obligatory that objects of different classes (location, month or season) could not be closer to each other than objects from the same classes. The *P* (Eq. 3) of Fig. 6c (perfect classification) is around the at the middle of the random and the best *P*-s. Our method is data driven and it helps to override traditional classification, where the data do not support to clearly perform classification.

Another example for the 2 objects – 1 variable sets case (RETSIM dataset) is shown in the Additional file 1.

### Seriation of neural network model data

Artificial neural network is one of the most popular methods to solve classification and regression tasks. The simplest conventional structure contains an input, a hidden and an output layer, where the input and the hidden ones and the hidden and the output ones are connected using sets of weights. In the simplest case, the hidden layer neurons contain activation functions, and the output layer ones only sum their weighted input. In the case of classification and regression, supervised method is used, where the weights are optimised to get a correct output for a training set. In an optimal situation, there is an independent test set to validate the model. There are two basic trends in the evaluation of models. In the novel applications of data science, we concentrate on the output performance without restricting the complexity of the ANN models. In the traditional case we would like to have limited complexity of the models with some possibility to interpret the model itself. The order of the neurons in the input, hidden and output layer are usually totally arbitrary. Therefore, it is an open question for seriation, especially, if we would like to interpret and understand a given model. Here we focus on some simple cases.

The first one is the visualisation of the weights between the input and the hidden layer. We might choose the hidden layer neurons as objects and the weights are the columns assigned to the input channels. The opposite assignment is also meaningful, where the input channels (input variables) are the objects, and the number of the variables is equal to the number of the neurons in the hidden layer. The same data matrix can be used, in one case the original matrix is seriated, in the other case its transpose is the input.

**Fig. 7:**
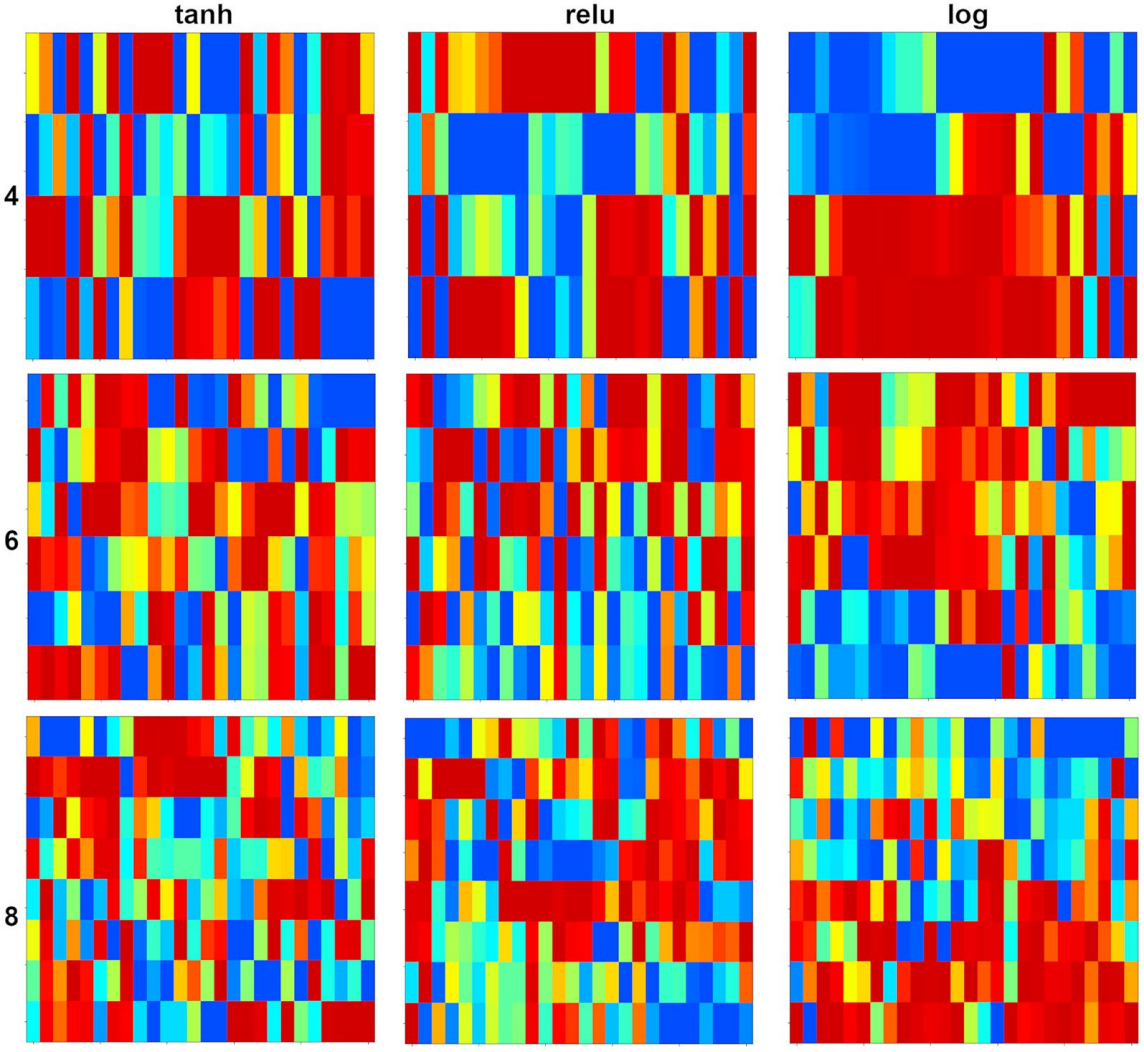
Seriated details of neural network models on the FLASHP1 dataset. The objects are the neurons, and the variables are the scaled weights of the original input variables. Three activation function are used (tangent hyperbolic, relu and logistic) with 4, 6 or 8 neurons in the hidden layer

In Fig. 7 we show the seriated results for the dataset FLASHP1. The original data was intended to estimate the flash point of different molecular systems. The predictor matrix contains information on the presence of different functional groups. We built several ANN models using several hyperparameters settings. Here we show 9 ANN models with three different activation functions and 4, 6 and 8 neurons in the hidden layer. The same training and test set was used for each model, and we selected models with both *R*^*2*^ and *Q*_*2*_^*F2*^ (*R*^*2*^_*test*_) more than 0.9. The graph shows the case, where the neurons were the objects, and the local variable spaces were formed by the weights assigned to the input channels. According to the calculation of the patch function (using Eq. 1), we scaled here the variables. It means, in the presence of both negative and positive weights blue colour might denote a large negative weight and red colour denotes large positive weight. Of course, the scaling might bias the interpretations, but in this feasibility study we do not intend to go really into the details of any ANN model. One can see that some of the seriated graphs (models with 4 neurons and models using logistic activation function) are clearly arranged providing the possibility of interpreting the operation of the model. In the case of this dataset, logistic activation seems to be the most interpretable group of models. We checked several high weight values at one-one neurons, and we assigned them as, e.g., F, O, or N containing functional groups. It means, these neurons are the responsible ones for different chemical parts as in ref. [48, 49]. This bunch of seriated graphs can be used to select models which are better interpretable.

**Fig. 8:**
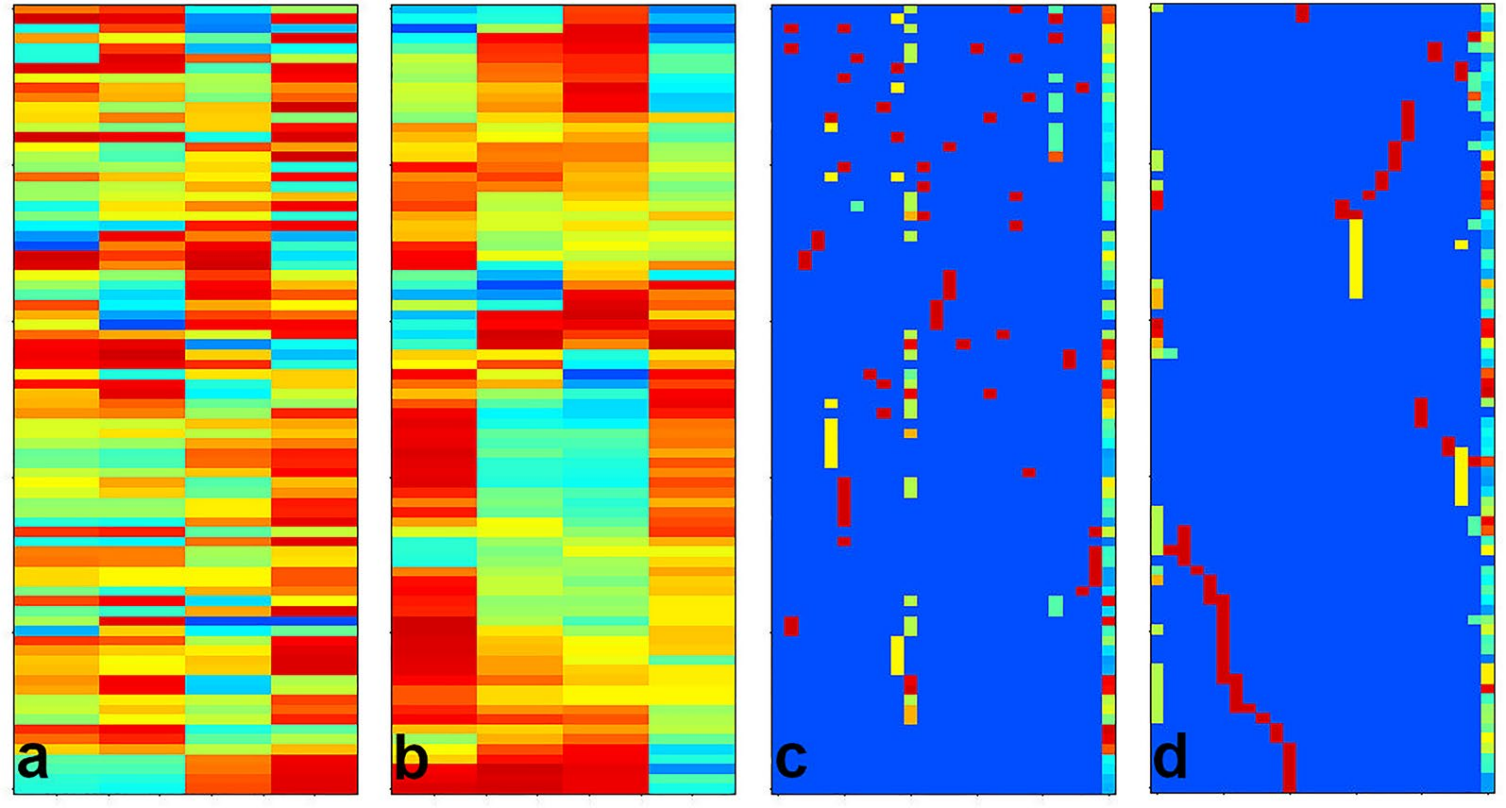
Seriation of the FLASHP1 data (test set). **a**–**b** Object – object activities on the hidden layer neurons (model: logistic function with 4 neurons) **a**- original **b**-seriated **c**–**d** Object – original variable data **c**-random order **d**-seriated

Our other example is the seriation of the objects (molecules) and their corresponding activity on the neurons as variables. One aspect of neural networks, that the original variables are mapped to the neurons of the hidden layer. This can be used as a dimensional reduction. Depending on the dimensionality of the original data and the number of the neurons, several features of the variables space might remain on the low-dimensional maps. A short investigation of it is shown in the Additional file 1 for hierarchical clustering. The object activities were calculated as the dot product of the variable vector of a molecule and the weight vector of a neuron in the hidden layer. Figure 8. shows a case, where 80 test objects are mapped on the neurons and molecules - original variables are shown, as well. One can see in Fig. 8a–b, that the patch seriation orders the molecules according to their activity on the neurons. This graph might be used to visually detect group of objects and details of the model, e.g., activity, inactivity, or redundancy of the models. This object activity -neuron seriation graph resembles somehow to unsupervised maps, e.g., a Kohonen map. The seriation in the original variable space is also successful, but here the variable space is 26 dimensional, while the neuron activity space is only 4 dimensional.

## Conclusions

We developed a new seriation method where our previous idea of using a global merit function based on a local quantity was improved. We defined a local similarity matrix containing the average similarity of neighbouring objects in a local 3-dimensional variable space. These local similarities were put into a global merit function, where the permutations of the object and variable vectors were directed to have both increased local similarities and forming patches of the large similarities.

The basic idea behind our seriation method is that there are datasets, where different parts of the variables are responsible for the different clusters of the objects. If a set of variables is not concerned in a cluster, they can be easily identified by being outside of the patches. In our method, an overlay contour plot of the local similarity values can be drawn onto the heatmaps of the original data to identify the clusters of the objects and the variables causing the clustering.

Both the local similarity matrix and the global patch function can generalize into more than two dimensions. We showed some examples of different three-dimensional cases, where the data were arranged according to two variable and one object axes or to one variable and two object axes. Furthermore, the local similarity and the patch function can be generalized for data with missing values or cases, where zero values need not be accounted as responsible ones for clustering.

We show two simulated datasets, where our patch method is especially effective to discover object clusters and the corresponding variables. Here the traditional seriation methods with non-local distances are mostly in trouble, the ad hoc values of the “non-important’ variables hinder the formation of the clusters. In the case of several public datasets, we found always clearly arranged heatmaps compared to the criss-crossed chequered random ones. Depending on the datasets (material science, compositional, air pollution, reaction kinetic data) clusters of objects and/or variables were always detectable in the seriated heatmaps. In the case of sparse matrices, the patch seriation glue together the non-zero variables.

In the case of three-dimensional seriation, the interpretation is less straightforward. One needs advanced three-dimensional graphical software or feasible two-dimensional maps to enhance visual perception. If the result is unfolded into two dimensions, the seriated data show periodic changes according to the dimensions merged visually into one axis. In our examples we show the details of three-dimensional air pollution data and retention of different mixtures on different columns.

We show some examples, how seriation helps to interpret neural network data. For example, we seriated the variable – hidden neuron weight matrices of different models and there is a striking difference depending on the activation function and the number of the neurons. For example, logistic activation function provided a more interpretable model than the other functions for a flash point dataset, especially at low number of hidden neurons. Also, seriation is a feasible method to detect the neurons responsible for a cluster of objects and to detect inactive parts of the models.

We think, that seriation is a powerful data evaluation or pre-evaluation method. Our special method forms patches of object and clusters. It is effective, if the non-important variables for a given cluster mask the identification possibility according to their variability. Seriation does not replace the different pattern recognition methods, but it at least helps to detect which methods and task might be successful on a dataset.

Up to now we seriated small- and medium-scale datasets. The highest number of objects was around 600 and the number of variables was 150. The feasibility of the method on large datasets needs new aims and justification, because the primary aim of seriation is to enhance visual interpretation on data heatmaps and the use of heatmaps has their limits for large datasets.

### Supplementary Information

**Additional file 1: Table S1.** Results of the thought experiment at different parameters. **Table S2.** Comparison of different seriation methods on the SIM dataset. **Figure S1.** 3-mode-3-way seriation of the FLASHP2 dataset. Projection of the highest three-dimensional local similarity matrix values on the two variable sets subspace. **Figure S2.** seriation of RETSIM data a) example of the simulated spectra b) 2-mode-2way seriation using retention intensities c) 3-mode-3-way seriation using retention intensities d) 2-mode-2-way seriation using retention intensities and fingerprints e) 3-mode-3way simulation using retention intensities and fingerprints. **Table S3.** Performance of seriation on the RETSIM dataset. **Figure S3.** Comparison of hierarchical clustering using all variables and neuron activities of the objects in dataset FLASHP2.

## Data Availability

The datasets supporting the conclusions of this article are available in the different repositories referenced one by one in Table 1. The C source code is deposited at [32].
