# Supplementary material for: Patch seriation to visualize data and model parameters
Source: J Cheminform. 2023 Sep 9;15:78. doi: 10.1186/s13321-023-00757-1 (PMC10492365; doi:10.1186/s13321-023-00757-1)
Supplement: Supplementary file 1 — Additional file 1: Table S1. Results of the thought experiment at different parameters. Table S2. Comparison of different seriation methods on the SIM dataset. Figure S1. 3-mode-3-way seriation of the FLASHP2 dataset. Projection of the highest three-dimensional local similarity matrix values on the two variable sets subspace. Figure S2. seriation of RETSIM data a) example of the simulated spectra b) 2-mode-2way seriation using retention intensities c) 3-mode-3-way seriation using retention intensities d) 2-mode-2-way seriation using retention intensities and fingerprints e) 3-mode-3way simulation using retention intensities and fingerprints. Table S3. Performance of seriation on the RETSIM dataset. Figure S3. Comparison of hierarchical clustering using all variables and neuron activities of the objects in dataset FLASHP2. [file 13321_2023_757_MOESM1_ESM.docx]

**Additional file material for**

Patch seriation to visualize data and model parameters

Rita Lasfar and Gergely Tóth*

Institute of Chemistry, Eötvös Loránd University

1117 Budapest, Pázmány sétány 1/a, Hungary

**1) Some merit and loss functions in seriation**

Here we summarize some basic seriation merit and loss functions using the study of [11] and references therein. The general formalism uses the term dissimilarity. The simplest dissimilarity matrix is the distance matrix of objects.

*A. Column/Row Gradient Measures:* A perfect anti-Robinsonian [26] matrix is a one mode dissimilarity matrix where the values only increase as they move away from the main diagonal.

within rows definition $d_{ik}\leq d_{ij} for 1\leq i<k<j\leq n$ Eq.S1

within columns definition $d_{kj}\leq d_{ij} for 1\leq i<k<j\leq n$ Eq. S2

In an anti-Robinsonian matrix the smallest dissimilarities are close to the diagonal. This provides a path to seriation. An appropriate merit function which indicates ‘divergence’ from the anti-Robinsonian was worked out by Hubert:

$M\left( D \right)= \sum_{i<k<j} f(d_{ik},d_{ij})+\sum_{i<k<j} f(d_{kj},d_{ij})$ Eq.S3

Where *f* is a function defining the violation or satisfaction of a gradient condition. There are several definitions for *f* in the literature.

*B. Hamiltonian Path Length* [11]*:* This function operates on the basis of presenting the dissimilarity matrix as a finite weighted graph G = (O,E) where O are the vertices (objects) and *e*_ij_ ∈ E represents the edges between the *i*-th and *j*-th object with the weight *w*_ij_ which represents *d*_ij_ in the dissimilarity. This graph can be used for seriation. Minimizing the Hamiltonian path (a path through which each node is visited only once) results in a seriation loss function through considering the dissimilarity between neighbouring objects:

$L\left( D \right)= \sum_{i=1}^{n-1} d_{i,i+1}$ Eq. S4

*C. Measure of Effectiveness:*

$M\left( X \right)= \frac{1}{2}\sum_{i=1}^{n} \sum_{j=1}^{m} x_{ij}[x_{i,j+1}+x_{i,j-1}+x_{i+1,j}+x_{i-1,j}]$ Eq.S5

It is maximized, if elements are closely related to their four neighbours. It should be noted that this equation can be separated into two parts for row and columns. This means that the merit functions for rows and columns are independent.

*D. Stress measures:* Compares the values in a matrix with their neighbours in a two-mode matrix. Two types are defined [25]:

The Moore neighbourhood: $\sigma_{ij}= \sum_{k=max(1,i-1)}^{min(n,i+1)} \sum_{l=max(1,j-1)}^{min(m,j+1)} {(x_{ij}-x_{kl})}^{2}$ Eq. S6

The Neumann neighbourhood: $\sigma_{ij}=\sum_{k=max(1,i-1)}^{min(n,i+1)} {(x_{ij}-x_{kj})}^{2} + \sum_{l=max(1,j-1)}^{min(m,j+1)} {(x_{ij}-x_{il})}^{2}$ Eq.S7

In the Neumann neighbourhood, unlike Moore’s, the influence of rows and columns are independent. In both cases a global stress measure can be built up:

$L\left( X \right)= \sum_{i=1}^{n} \sum_{j=1}^{m} \sigma_{ij}$ Eq.S8

**2) Thought experiment to show that the formation of patches increases the patch function.**

Let us suppose and *NxN* local similarity matrix containing N/2 s_1_ and s_2_ values. If *N* is large enough, the probability of having a neighbouring *s_1_* or *s_2_* is 0.5 and 0.5. In the case of a random arrangement, the expectation value of the patch function (Eq.3) is *P(random)=2(N-1)(N-1)(1/4(s_1_s_1_)q+1/4(s_2_s_2_)q+1/2(s_1_*s_2_)q)*. If *s_1_* and *s_2_* values are ordered that *s*_1_ is on one side and *s*_2_ is on the other side of the matrix, than *P(ordered)=2(N2-1)(N-1)((s_1_s_1_)q+(s_2_s_2_)q)+2(N-1)(s_1_s_2_)q*.

Additional file Table S1: Results of the thought experiment at different parameters

| *N* | *s_1_* | *s_2_* | *q* | *P(random)* | *P(ordered)* |
| --- | --- | --- | --- | --- | --- |
| 100 | 0.4 | 0.6 | 2 | 1.35 10^7^ | 1.55 10^7^ |
| 100 | 0.4 | 0.6 | 3 | 3.92 10^6^ | 5.07 10^6^ |
| 100 | 0.1 | 0.9 | 2 | 3.36 10^7^ | 6.56 10^7^ |
| 100 | 0.1 | 0.9 | 3 | 2.66 10^7^ | 5.31 10^7^ |
| 500 | 0.4 | 0.6 | 2 | 8.45 10^9^ | 9.70 10^9^ |
| 500 | 0.4 | 0.6 | 3 | 2.45 10^9^ | 3.17 10^9^ |
| 500 | 0.1 | 0.9 | 2 | 2.10 10^10^ | 4.10 10^10^ |
| 500 | 0.1 | 0.9 | 3 | 1.67 10^10^ | 3.32 10^10^ |

**3) Calculation of local similarity array and patch function in three dimensions**

*1-object–2-variable sets case*

If we have a two-dimensional *n×m* data matrix, where the columns contain two separable set of variables, we might perform a seriation, where the order of the objects, the first set of variables and the second set of variables can be separately sequenced. The number of the objects is *n* and the number of the columns is *m=ma+mb*. *ma* is the number of the first set of variables and *mb* belongs to the second set. *1≤ja≤ma* and *1≤jb≤mb* are the indices of the variables, but the second set of variables are stored in the *ma+jb*-th columns. A three-dimensional local similarity array can be constructed, where an element shows the average similarity of the given object to its neighbours (axis one), but now in two local three-dimensional variable spaces (axis two or axis three). Using the first type of variables and the second type of variables independently, we calculate the two local similarities between the given object and one of its neighbours. The final *s*_i,ja,jb_ local similarity contains the average of four similarities (over the two neighbours times the two local variable spaces).

${la}_{i,k,ja}= \sqrt{\sum_{l=ja-1}^{ja+1} \left( \frac{a_{kl}-a_{il}}{{diff}_{max,l}} \right)^{2}}$ Eq.S9

${lb}_{i,k,jb}= \sqrt{\sum_{l=ma+jb-1}^{ma+jb+1} \left( \frac{a_{kl}-a_{il}}{{diff}_{max,l}} \right)^{2}}$ Eq.S10

$s_{i,ja, jb}=0.5\left( \sum_{k=i-1,i+1} 1- \frac{{la}_{i,k,ja}}{D_{col,ja}} \right)/D_{row,i}+0.5\left( \sum_{k=i-1,i+1} 1- \frac{{lb}_{i,k,jb}}{D_{col,jb}} \right)/D_{row,i}$ Eq.S11

,where *la_ikja_* and *lb_ikjb_* are their local distances in the variable space formed separately by the first and second set of variables. *k* takes only the values *i-1* and *i+1* for a given *i* index of objects. *diff*_max,l_ is the difference between the largest and the smallest elements of the *l*-th column in *A*. It is used to scale the distance between [0,sqrt(3)], if the local variable space contains three variables. If the *ja*-th or *jb*-th variable is at the first or the last column of the given variable set, the local space contains only [0,sqrt(2)]scaled distances. *D_col,ja_*  and *D_col,jb_*  contain the corresponding upper bounds of the intervals for each variable. *D_row,I_* is usually two for the *i*-th object except the first and the last rows, where it is one. These row or column dependent quantities (*diff_maxl_, D_col,j_, D_row,i_*) were introduced to be able to get theoretically *s*_i,ja,jb_ ϵ [0,1] for all positions including the non-bulk matrix elements.

In the case of missing data or zero data to be omitted, the local similarity array reads as:

$s_{i,ja,jb}=\sum_{k=i-1,i+1} \sum_{l=ja-1}^{ja+1} \left( 1-\left| \frac{a_{kl}-a_{il}}{{diff}_{max,l}} \right| \right)/12+\sum_{k=i-1,i+1} \sum_{l=ma+jb-1}^{ma+jb+1} \left( 1-\left| \frac{a_{kl}-a_{il}}{{diff}_{max,l}} \right| \right)/12$ Eq. S12

Here the sums are skipped, if any of the data is missing or zeros are intended to be skipped. using this definition, the local similarities cannot be one at the borders or if they are involved in any skip.

*2-objects–1-variable sets case, original data in two dimensions*

An *n×m* two-dimensional data matrix may contain two different sets of objects. The number of the rows is *n=na+nb*. *na* is the number of the first set of objects and *nb* belongs the second set. *1≤ia≤na* and *1≤ib≤nb* are the indices of the objects, but the second set of objects are in the *na+ib* rows. The two sets of objects might be seriated independently. Here the first axis of the local similarity matrix contains the first set of objects, the second one is the second set of objects and the third axis shows the variables. The *s*_ia,ib,j_ local similarity contains the average of 4 similarities: the similarity of the *ia-(ia-1)* and *ia-(ia+1)* object pairs of the first axis and the *ib-(ib-1)* and *ib-(ib+1)* object pairs of the second axis. The local variable space for this element is spanned by the *j-1, j, j+1* variables. *k* takes only the values *ia-1* and *ia+1* or *ib-1* and *ib+1* for a given *ia* or *ib* index of objects.

${la}_{ia,k,j}= \sqrt{\sum_{l=j-1}^{j+1} \left( \frac{a_{kl}-a_{ia,l}}{{diff}_{max,l}} \right)^{2}}$ Eq.S13

${lb}_{ib,k,j}= \sqrt{\sum_{l=j-1}^{j+1} \left( \frac{a_{na+k,l}-a_{na+ib,l}}{{diff}_{max,l}} \right)^{2}}$ Eq.S14

$s_{ia,ib, j}=0.5\left( \sum_{k=ia-1,ia+1} 1- \frac{{la}_{ia,k,j}}{D_{col,j}} \right)/D_{row,ia}+0.5\left( \sum_{k=ib-1,ib+1} 1- \frac{{lb}_{ib,k,j}}{D_{col,j}} \right)/D_{row,ib}$ Eq.S15

,where *la_ia,k,j_* and *lb_ib,k,j_* are their local distances in the variable space. *D_row,Ia_*  and *D_row,Ib_*  are usually two for the objects except the first and the last rows separately for the sets, where it is one. *s*_ia,jb,j_ ϵ [0,1] values for all positions including the non-bulk matrix elements.

In the case of missing data or zero data to be omitted, the local similarity array reads as:

$s_{ia,ib,j}=\sum_{k=ia-1,ia+1} \sum_{l=j-1}^{j+1} \left( 1-\left| \frac{a_{kl}-a_{ia,l}}{{diff}_{max,l}} \right| \right)/12+\sum_{k=ib-1,ib+1} \sum_{l=j-1}^{j+1} \left( 1-\left| \frac{a_{na+k,l}-a_{na+ib,l}}{{diff}_{max,l}} \right| \right)/12$ Eq. S16

Here the sums are skipped, if any of the data is missing or zeros are intended to be skipped. Using this definition, the local similarities cannot be one at the borders or if they are involved in any skip.

*2-objects–1-variable sets, original data in three dimensions*

An *na×nb×m* three-dimensional data matrix may contain two different sets of objects in the first two dimensions. *na* is the number of the first set of objects and *nb* belongs the second set. *1≤ia≤na* and *1≤ib≤nb* are the indices of the objects. The first axis of the local similarity matrix contains the first set of objects, the second one is the second set of objects, and the third axis shows the variables. The *s*_ia,ib,j_ local similarity contains the average of 4 similarities: the similarity of the *ia-(ia-1)* and *ia-(ia+1)* object pairs of the first axis and the *ib-(ib-1)* and *ib-(ib+1)* object pairs of the second axis. The local variable space for this element is spanned by the *j-1, j, j+1* variables, but their value depends both on the actual *ia* and *ib* indices. *k* takes only the values *ia-1* and *ia+1* or *ib-1* and *ib+1* for a given *ia* or *ib* index of objects.

${la}_{ia,ib,k,j}= \sqrt{\sum_{l=j-1}^{j+1} \left( \frac{a_{k,ib,l}-a_{ia,ib,l}}{{diff}_{max,l}} \right)^{2}}$ Eq.S17

${lb}_{ia,ib,k,j}= \sqrt{\sum_{l=j-1}^{j+1} \left( \frac{a_{ia,k,l}-a_{ia,ib,l}}{{diff}_{max,l}} \right)^{2}}$ Eq.18

$s_{ia,ib, j}=0.5\left( \sum_{k=ia-1,ia+1} 1- \frac{{la}_{ia,ib,k,j}}{D_{col,j}} \right)/D_{row,ia}+0.5\left( \sum_{k=ib-1,ib+1} 1- \frac{{lb}_{ia,ib,k,j}}{D_{col,j}} \right)/D_{row,ib}$ Eq.S19

,where *la_ia,k,j_* and *lb_ib,k,j_* are their local distances in the variable space. *diff*_max,l_ is the difference between the largest and the smallest elements of the *l*-th variable in *A*. *s*_ia,jb,j_ ϵ [0,1] values for all positions including the non-bulk matrix elements.

In the case of missing data or zero data to be omitted, the local similarity array reads as:

$s_{ia,ib,j}=\sum_{k=ia-1,ia+1} \sum_{l=j-1}^{j+1} \left( 1-\left| \frac{a_{k,ib,l}-a_{ia,ib,l}}{{diff}_{max,l}} \right| \right)/12+\sum_{k=ib-1,ib+1} \sum_{l=j-1}^{j+1} \left( 1-\left| \frac{a_{ia,k,l}-a_{ia,ib,l}}{{diff}_{max,l}} \right| \right)/12$ Eq. S20

Here the sums are skipped, if any of the data is missing or zeros are intended to be skipped. Using this definition the local similarities cannot be one at the borders or if they are involved in any skip.

*Calculation of the patch function in three dimensions*

$P= \sum_{i=1}^{n-1} \sum_{j=1}^{m} \sum_{k=1}^{l} {2\left( s_{ijk}s_{i+1,jk} \right)}^{q}+\sum_{i=1}^{n} \sum_{j=1}^{m-1} \sum_{k=1}^{l} {2\left( s_{ijk}s_{i,j+1,k} \right)}^{q}+\sum_{i=1}^{n} \sum_{j=1}^{m} \sum_{k=1}^{l-1} {2\left( s_{ijk}s_{ij,k+1} \right)}^{q}$ Eq S21

where S is the *n×m×l* three-dimensional local similarity matrix and *q* is the power exponent (usually set to 2 or 3).

**4) Comparison of the patch method to other seriation methods accessible in the seriation package of R.**

Details of the methods can be found in the article of Hahsler et al. [11]. The SIM dataset was used (50 objects, 20 variables), where the set was ordered in 4 clusters of 10 objects each and 10 random objects not associated with any clusters. There were two overlapping variables for clusters C and D. Optimally, the 4 object clusters and the corresponding variables are seriated into one-one group. In the case of distance matrix inputs, there were two seriations. One was on the object distance matrix to get number of the object groups, and one was performed on the ‘distance matrix’ of the variables to get variable groups. Numbers higher than 4 means that groups are separated into more parts.

Additional file Table S2. Comparison of different seriation methods on the SIM dataset

| **Keyword in R** | **Short description** | **No. of object groups** | **No. of variable groups** |
| --- | --- | --- | --- |
| Theoretical |  | **4** | **4** |
| Random |  | 33 | 18 |
| *input: 2D matrix with objects in rows and variables in columns* | | | |
| Patch (in our C code) | patch function on local similarity matrix opt. in Monte Carlo sim. | **4** | **4** |
| BEA | measure of effectiveness, opt. by bond energy algorithm | 26 | 18 |
| BEA_TSP | measure of effectiveness, opt. by travelling salesman algorithm | 20 | 16 |
| PCA | least square crit. opt. in principal comp. analysis | 27 | 14 |
| PCA_angle | variant of PCA | 19 | 13 |
| *input: distance matrix separatley for objects and variables (two independent seriations)* | | | |
| ARSA | linear. seriation crit. opt. by simulated annealing | 19 | 17 |
| TSP | hamiltonian path length opt. by TSP solver | 21 | 15 |
| R2E | rank-two ellipse seriation | 22 | 14 |
| MDS | least square crit. | 27 | 14 |
| MDS_metric | MDS variant | 27 | 14 |
| MDS_nonmetric | MDS variant | 22 | 14 |
| MDS_angle | MDS variant | 19 | 13 |
| HC | hierarchial clustering with different linkages | 28 | 17 |
| HC_single | HC with different linkage | 33 | 16 |
| HC_complete | HC with different linkage | 28 | 17 |
| HC_average | HC with different linkage | 25 | 16 |
| HC_ward | HC with different linkage | 24 | 17 |
| GW | hamiltonian length opt. by Gruvaeus and Wainer algorithm | 26 | 16 |
| OLO | hamiltonian length opt. by optimal leaf ordering | 28 | 13 |
| VAT | visual assessment of clustering tendency method | 29 | 16 |
| Spectral | 2-sum criterion with spectral seriation | 32 | 16 |
| Spectral_norm | variant of Spectral | 24 | 15 |
| SPIN_NH | sorting points into neighbourhoods method | 21 | 18 |
| SPIN_STS | variant of SPIN_NH | 30 | 18 |
| QAP_LS | linear seriation criterion opt. by quadratic assignment formulation | 17 | 16 |
| QAP_2SUM | 2-sum crit. opt. by QAP method | 29 | 14 |
| QAP_BAR | banded AR form opt. by QAP method | 24 | 13 |
| QAP_Inertia | inertia crit. opt. by QAP method | 22 | 14 |

**5) Projection of 3D seriation – FLASHP2 dataset**

As an example of possible projections of the three-dimensional local similarity matrix we show here the projection of the highest values on the two variable sets subspace for FLASHP2 dataset. The corresponding seriations are discussed in the main manuscript and shown in Fig. S1 is the projection, where the highest local similarities over the objects are collected for a given set one – set two variable pair. It shows that the highest similarities are obtained for which local variable pairs of set one and set two ones.


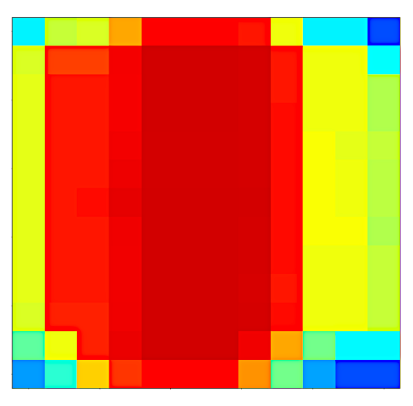


Figure S1. 3-mode-3-way seriation of the FLASHP2 dataset. Projection of the highest three-dimensional local similarity matrix values on the two variable sets subspace.

**6) List of row and column names in Table 2 in the manuscript**

Row names (cities at countryside): 1-Székesfehérvár, 2-Tatabánya, 3-Eger, 4-Kecskemét, 5-Veszprém, 6-Szombathely, 7-Sopron, 8-Pécs, 9-Debrecen, 10-Nyiregyháza, 11-Szolnok, 12-Győr, 13-Miskolc, 14-Szeged; Columns (locations in Budapest): 1-Budatétény, 2-Csepel, 3-Pesthidegkút, 4-Gilice tér, 5-Káposztásmegyer, 6-Kőrakás, 7-Kosztolányi Dezső tér, 8-Teleki tér, 9-Erzsébet tér, 10-Széna tér, 11-Gergely utca, 12-Honvéd

**7) 3D seriation of the RETSIM dataset**

Similarly to the three-dimensional seriation of the POL-MONTH dataset in the main manuscript, we show here another case of two dependent object sets - one variable set situation. The data are originally three dimensional, but we usually have an unfolded two-dimensional data matrix. In the case of the RETSIM data unfolded to two dimensions, the first six rows correspond to the retention times of the 6 mixtures on the first chromatographic column, the next six rows for the second column, etc… The variables (columns) were the retention intensities for 1-100 arbitrary time intervals. The three axes of the local similarity matrix could be the chromatographic columns, the mixtures and the retention times. The *s*_ijk_ local similarity contains the average of 4 similarities: the similarity of the *j-th* mixture data for the *i-(i-1)* and *i-(i+1)* chromatographic column pairs and the similarity of the *i-th* chromatographic column data for the *j-(j-1)* and *j-(j+1)* mixture pairs. The local variable space for this element is spanned by the *k-1, k, k+1* retention time intensities.

If we seriate the three axes independently (only the order of the columns and separately the order of the mixtures), a rather limited information can be obtained on the new order, e.g., some link between the mixtures and columns. Furthermore, for such a seriation we need to know a priori the mixture and column labels for each spectrum. It would be more interesting, if we seriate in an unrestricted way, where we fill the 6 times 6 object space with maximal freedom without taking care on the order of the columns and the mixtures. In this case, seriation has a strong explanatory statistical feature, if we will be able, e.g., to cluster the common measurements for a column or a given mixture.

The simple 2-mode-2-way seriation of the retention spectra was able to classify the spectra for each column perfectly (Fig. S2b). On contrary, there were no patterns how the mixtures were ordered. If we use the three-dimensional spectra with supposing a 6x6x100 local similarity matrix, we still obtained a good order for the columns (Fig. S2c), but the interpretation of the result is not easy. In the three-dimensional seriation an object has two-two neighbours in two variable subspaces with distances at three local retention times. There is a chance distribution that which type of objects are neighbouring in which spaces, e.g., there is no driving force that the column like neighbouring is according to the first axis or the second one. Even more, it can be different at the different grids of the local similarity matrix. For example, the three-dimensional seriation provided a good arrangement for the columns, around 70 % of the 4 neighbours were measurements on the same columns. Spatially, it occurred along two axes and the unfolded data were partitioned into 3-3 long sequences for each column. It is not easy to see it on the unfolded seriated data.

We found that it is not straightforward how to seriate the mixtures using the retention times. Therefore, we added 50 more variables to the data, where the largest 50 data of a row was normalized with the largest in the same row and were sorted in a descending order. These 50 new variables work as mixture specific fingerprints. We performed 2-mode-2-way and 3-mode-3-way seriations using this extended variable set. The results are shown in tabular form over Fig. S2 d-e, since the three-dimensional seriations cannot be unfolded by a visually striking way (c.f. the unambiguity of the assignment of the planes of the ordering and the column-mixture features.) Some features of the seriation are shown in Table S3. It can be seen that the use of three-dimensional local similarity matrix helped slightly to get better classification on the mixtures, as well. 100 (84+16) or 106(84+22) correct neighbours were sorted from the altogether 120 possible neighbouring positions.





Figure S2. seriation of RETSIM data a) example of the simulated spectra b) 2-mode-2way seriation using retention intensities c) 3-mode-3-way seriation using retention intensities d) 2-mode-2-way seriation using retention intensities and fingerprints e) 3-mode-3way simulation using retention intensities and fingerprints

Table S3. Performance of seriation on the RETSIM dataset

| Seriation type | Maximal theoretical number of correct column neighbours + correct mixture neighbours (2D), or of all neighbours (3D) | Realization in an example for columns | Realization in an example for mixtures |
| --- | --- | --- | --- |
| 36x100 size, 2D | 60+60 | 60 (perfect classification) | 0 |
| 36x150 size, 2D | 60+60 | 60 (perfect classification) | 0 |
| 6x6x100 size, 3D | 120 | 84 (3-3 groups in 2D unfolded map) | 16 |
| 6x6x150 size, 3D | 120 | 84 (3-3 groups in 2D unfolded maps) | 22 (repetition of mixtures in +-6 shifts) |

**8) Hierarchical clustering of objects using neuron activities**

The following short investigation shows that hierarchical clustering using all original variables can be replaced by using the neuron activities of objects. The crucial point is the determination of the number of the hidden neurons. 25 test objects of ANN models on the FLASHP2 dataset with molecular descriptors (13 variables) are clustered on models with different number of hidden layer neurons (4,6,8,10). It can be seen in Fig. S3 that clustering of the ANN models approximates the grouping of all data case with increasing number of hidden neurons. The most significant clusters are well separated at all sizes. For the other clusters the 4 neurons seem to be slightly different from the original model, while 6-8-10 neuron ones provide qualitatively more and more similar patterns for many of the clusters.

In the case of the FLASHP1 dataset, the clustering based on the 26 original variables was partially reproduced in all the 4, 6, 8, 10 hidden layer neuron models. It means, some highly compact or well-separated clusters were close to identical at each hidden layer size (concerning around half of the objects), while the clustering of the other objects did not approximate systematically the all-data model with increasing number of neurons.

We performed hierarchical clustering on a dataset not used directly in seriation. This was the cetane number estimation dataset of Saldana et al. [29,36] published together with FLASHP2. Here, from 8 hidden layer neurons the clustering of all-variable case (15 variables) was well reproduced, but the 4 and 6 neuron variants provided very limited similarity in the dendograms.


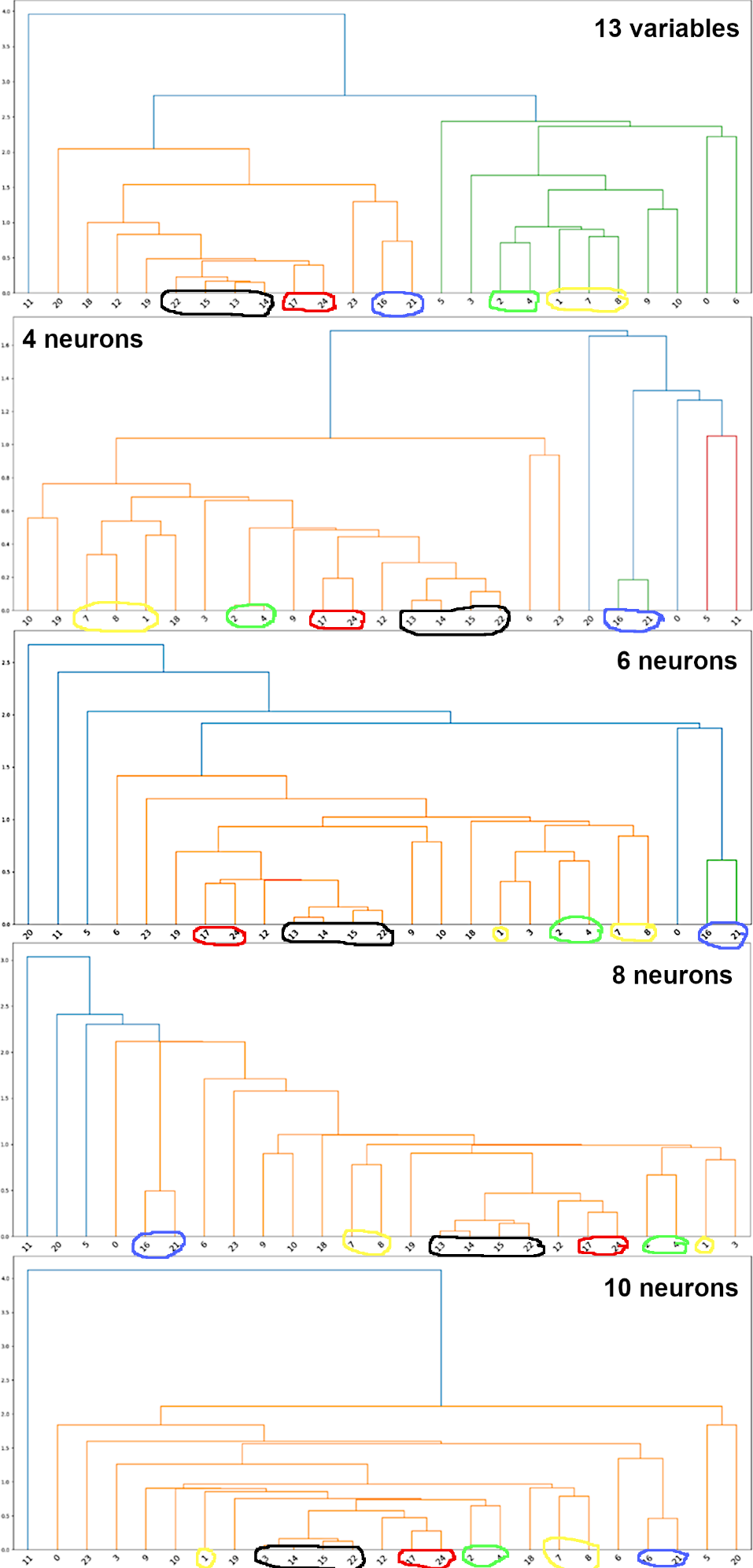


Figure S3. Comparison of hierarchical clustering using all variables and neuron activities of the objects. Dataset FLASHP2.
